# Assessing the causal associations of sleep apnea with mental health and socioeconomic status: a bidirectional two-sample Mendelian randomization

**DOI:** 10.1186/s12920-023-01783-6

**Published:** 2024-01-22

**Authors:** Yuan Wu, Zuming Li, Xueru Chen, Siyi Wu, Xuemei Zhong, Aifang Zheng, Li Li, Hai Chen, Jiqiang Li, Yue Lu, Jiankun Chen, Kao Gan

**Affiliations:** 1 The Second Clinical Medical College, Guangzhou University of Chinese Medicine, Guangzhou, China; 2 The Second Affiliated Hospital of Guangzhou, University of Chinese Medicine (Guangdong Provincial Hospital of Chinese Medicine), Guangzhou, China; 3 Kashgar First People’s Hospital, The Xinjiang Uygur Autonomous Region, Kashgar, China

**Keywords:** Sleep apnea, Mental health, Socioeconomic status, Mendelian randomization

## Abstract

**Objective:**

Traditional observational research has suggested a connection between socioeconomic position, mental health, and sleep apnea (SA), but the specifics of this connection are still unclear. Using the Mendelian randomization approach, we intended to evaluate the potential causal link between mental health, socioeconomic status, and SA.

**Methods:**

Our research employed summary statistics data from large-scale genome-wide association studies (GWAS) on mental health, socioeconomic status, and SA. In the main study, the connection between mental health, socioeconomic status, and SA was examined using the inverse variance weighted approach. In addition, as a supplement, we also used other Mendelian randomization methods, including MR Egger, weighted median, simple mode, and weighted mode.

**Results:**

The primary analysis showed that educational attainment, including longer years of schooling, college or university degree, and higher intelligence was associated with a lower risk of SA (OR = 0.750, 95%CI = 0.653–0.862; OR = 0.558, 95%CI = 0.423–0.735; OR = 0.871, 95%CI = 0.760–0.999, respectively), while social deprivation was associated with a higher risk of SA (OR = 1.821, 95%CI = 1.075–3.085). And the income was not associated with the risk of sleep apnea (OR = 0.877, 95%CI = 0.682–1.129). In mental health exposure, major depressive disorder was associated with a higher risk of sleep apnea (OR = 1.196, 95%CI = 1.015–1.409), while attention-deficit hyperactivity disorder, bipolar disorder, and schizophrenia were not associated with the risk of sleep apnea (OR = 1.064, 95%CI = 0.958–1.181; OR = 1.030, 95%CI = 0.942–1.127; OR = 0.990, 95%CI = 0.957–1.025, respectively). Reverse MR analysis failed to find a causal effect from SA on mental health and socioeconomic status.

**Conclusions:**

This MR investigation offers proof of a possible causal relationship between SA, socioeconomic level, and mental health.

**Supplementary Information:**

The online version contains supplementary material available at 10.1186/s12920-023-01783-6.

## Background

Sleep apnea (SA), the most widespread form of sleep-disordered breathing, is defined by the repeated partial or total closure of the upper airway during sleep [1]. Previous research revealed a strong relationship between SA and socioeconomic level and mental health. In contrast to the general population, patients with mental health disorders had a greater incidence of SA, because patients with severe mental illness are frequently prescribed psychotropic medications, which can result in weight gain and metabolic syndrome, both of which are known risk factors for SA, as well as sedative medications interrupt sleep architecture [2]. And vice versa, patients with SA had a considerably higher prevalence of mental health issues than control individuals [2–8], including attention-deficit hyperactivity disorder (ADHD), bipolar disorder, major depressive disorder, schizophrenia, and anxiety disorders. In addition, having a higher degree of education was linked to a decreased risk of SA, whereas people with a poor socioeconomic status have a higher risk of SA [9, 10]. Given the promiscuous variables and reverse causation that are inherent in observational studies, the connection between mental health, socioeconomic position, and SA is yet unknown and calls for more research.

Mendelian randomization (MR) is an innovative analysis that uses genetic variations as instrumental variables (IV) to evaluate the causality of an observed association between a modifiable exposure and a clinically relevant outcome [11]. Because genotypes are randomly assigned to offspring, the association between genetic variants and outcome is unaffected by common confounders, and a causal sequence is plausible [12]. Furthermore, because genetic variation occurs before disease and the order of the two cannot be reversed, MR can avoid the interruption of reverse causality [13]. Although there is growing evidence that MR is reliable, there are few studies that focus on the relationship between mental health, socioeconomic status, and SA. As a result, we performed a two-sample bidirectional MR study to examine the causal association between mental health (including ADHD, bipolar disorder, major depressive disorder, schizophrenia, and anxiety disorders), socioeconomic status (including educational attainment, income, and social deprivation), and SA. Although the results’ generalizability may be limited by race, this study may help to reveal the genetic characteristics of mental health, socioeconomic status, and SA through MR analysis, which actively contributes to the further study of SA.

## Method

### Data sources

**Fig. 1:**
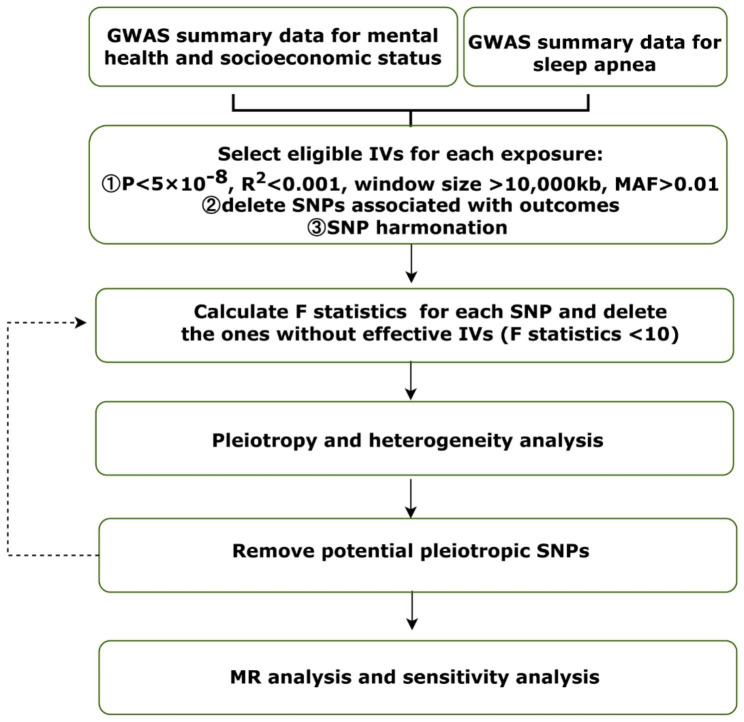
Flowchart of MR analysis in this study. GWAS, genome-wide association studies; IV, instrumental variables; MAF, minor allele frequency; SNPs, single-nucleotide polymorphisms; MR analysis, Mendelian randomization analysis

The study design is simply described in a flowchart (Fig. 1). Educational attainment is assessed by years of schooling, college or university degree, and intelligence. For summary statistics for mental health and socioeconomic status, we used data from the Social Science Genetic Association Consortium (SSGAC) [14], the UK Biobank (UKB) [15], the Psychiatric Genomics Consortium (PGC), and Savage, et al. (2018) [16], as shown in Table 1. The SSGAC is a collaborative effort between social scientists and medical researchers that conducts genetic association studies for social science outcomes and offers a forum for multidisciplinary cooperation and idea-sharing. The UKB project is a sizable prospective cohort study with around 500,000 participants from the United Kingdom. The PGC, which leverages the power of more than 900,000 individuals, is the biggest biological study in the history of psychiatry.

We used large-scale, publicly accessible genome-wide association studies (GWAS) data from the FinnGen research project to construct a summary statistic for sleep apnea [17], which included 16,761 cases and 201,194 control subjects.

**Table 1:** Summary of genome-wide association studies used in the analyses

Phenotype	Source	Sample size, n			SNPs, n
		Total	Cases	Controls	
Years of schooling	SSGAC	766,345	-	-	317
College or university degree	UKB	334,070	106,305	227,765	188
Intelligence	Savage, et al.	269,867	-	-	165
Income	UKB	397,751	-	-	48
Social deprivation	UKB	462,464	-	-	18
ADHD	PGC	55,374	20,183	35,191	12
Bipolar disorder	PGC	51,710	20,352	31,358	16
Major depressive disorder	PGC	480,359	135,458	344,901	36
Schizophrenia	PGC	320,404	76,755	243,649	217
Anxiety disorders	UKB	361,194	1,092	360,102	0
Sleep apnea	FINN	217,955	16,761	201,194	5

### Genetic instrumental variables (IV) selection

Based on the GWAS summary data on socioeconomic status and mental health, we chose the appropriate genetic IVs using several quality control criteria. First, for each exposure, we utilized independent genetic variants that were strongly related (P < 5 × 10^− 8^). To prevent linkage disequilibrium (LD), we then carried out the clumping method with R^2^ < 0.001 and a window size > 10,000 kb [18]. Third, SNPs having a minor allele frequency (MAF) of less than 0.01 were disregarded. We looked for every single-nucleotide polymorphism (SNP) included in our research and eliminated SNPs linked to SA. To correct the alleles’ orientation, SNP harmonization was also conducted [19].

### Evaluation of the strength of the genetic instruments

To evaluate the strength of genetic instruments for various forms of mental health and socioeconomic status, we calculated the F statistics for each SNP. IVs with F statistics less than 10 were considered weak instruments, and the exposure would not be included in MR analysis [20].

### Pleiotropy, heterogeneity, and sensitivity analysis

The probability of horizontal pleiotropy was evaluated using MR-Egger regression, as shown by the intercept [21]. MR Pleiotropy Residual Sum and Outlier (MR-PRESSO) package in R was used to detect pleiotropy (P < 0.05), and the SNP with the smallest pleiotropy P value was eliminated [22]. The heterogeneity was also identified using the inverse variance weighted (IVW) method and MR-Egger regression, and Cochrane’s Q statistics were employed to quantify it. To determine the stability of the results, we also performed the leave-one-out analysis.

### MR analysis

The wald estimates of causation for each IV are combined using the IVW technique using a meta-analysis methodology, yielding the best accurate estimates with balanced pleiotropy. To reduce the impact of single SNPs on phenotypes, the pleiotropic and causal effects were detected in this study using the MR-Egger approach, when more than half of the chosen SNPs were legitimate genetic variations. Although MR-Egger can identify and modify pleiotropy, the estimation accuracy produced by this method is extremely low [23]. Weighted median gives a trustworthy impact estimate of the causal influence based on the assumption that at least half of IVs are valid, enhancing precision and robustness [24]. Although simple mode is not as impactful as IVW, it could provide pleiotropy robustness [25]. Weighted mode is susceptible to mode estimation’s difficult bandwidth selection [26]. The simple mode and weighted mode can also be applied to estimate the causal effect [27]. When compared to other MR approaches, the IVW method’s statistical effectiveness was noticeably greater [28], thus, IVW data were prioritized in this investigation to determine the causal relationships between exposures and the risk of SA. In addition, we performed MR-Egger, weighted median, simple mode, and weighted mold as complementary methods. The MR analysis was performed in R software (version 4.2.2) using the TwoSampleMR package.

## Results

### The causal effect of mental health and socioeconomic status on SA (fig. 2)

Anxiety disorders were excluded in the analysis of causal effects from mental health and socioeconomic status to SA since there were no effective IVs. Overall, mental health exposures including ADHD, bipolar disorder, major depressive disorder, and schizophrenia, and socioeconomic status exposures including years of schooling, college or university degree, intelligence, income, and social deprivation were included in our study. The number of SNPs for each exposure varies from 9 to 305 after a series of quality control steps (Table 2, Supplementary Tables 1–10). The F statistic suggests that the SNP is a strong instrument.

MR estimates of different methods are presented in Table 2. Since the analyses of mental health and socioeconomic status showed significant heterogeneity but no directional pleiotropy, the multiplicative random-effect models were applied in the IVW analysis, and four causal associations from five socioeconomic statuses to sleep apnea were identified, while only one causal association from four mental health exposures was observed for sleep apnea. We found evidence that educational attainment, including longer years of schooling, college or university degree, and higher intelligence was associated with a lower risk of sleep apnea (OR = 0.750, 95%CI = 0.653–0.862; OR = 0.558, 95%CI = 0.423–0.735; OR = 0.871, 95%CI = 0.760–0.999, respectively), while social deprivation was associated with a higher risk of sleep apnea (OR = 1.821, 95%CI = 1.075–3.085). The income was not associated with the risk of sleep apnea (OR = 0.877, 95%CI = 0.682–1.129). In mental health exposure, major depressive disorder was associated with a higher risk of sleep apnea (OR = 1.196, 95%CI = 1.015–1.409), while ADHD, bipolar disorder, and schizophrenia were not associated with the risk of sleep apnea (OR = 1.064, 95%CI = 0.958–1.181; OR = 1.030, 95%CI = 0.942–1.127; OR = 0.990, 95%CI = 0.957–1.025, respectively). The results of the MR-Egger intercept indicated that there was no pleiotropic effect (P > 0.05). However, MR-PRESSO detected outliers, revealing that the IVs of years of schooling, college or university degree, and intelligence and outcome had a substantial horizontal pleiotropy (P < 0.05). Results did not change substantially the after removal of outliers (Table 3). The results from the leave-one-out analyses indicated that the causal effect of mental health and socioeconomic status on SA was stable.

The scatter plots, forest plots, and funnel plots for sleep apnea are displayed in complementary materials (Supplementary Figs. 1–3).

**Fig. 2:**
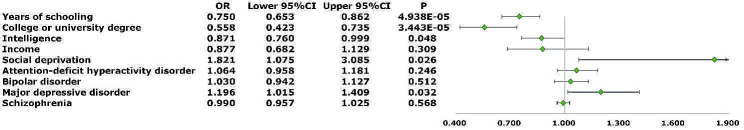
The causal effect of mental health and socioeconomic status based on the IVW method. OR, odds ratio; CI, confidence interval; IVW, inverse variance weighted

**Table 2:** Results of the MR study testing causing the association between risk factors and sleep apnea

Analysis	OR	Lower 95%CI	Upper 95%CI	P	SNPs, n	Horizontal pleiotropy		Heterogeneity	
						Egger intercept	P	Q	P
**Years of schooling**									
MR Egger	0.610	0.354	1.050	0.076	305	0.003	0.441	415.781	2.072E-05
Weighted median	0.747	0.621	0.898	0.002					
Inverse variance weighted	0.750	0.653	0.862	4.938E-05					
Simple mode	0.769	0.424	1.396	0.389					
Weighted mode	0.709	0.436	1.153	0.166					
**College or university degree**									
MR Egger	0.485	0.142	1.662	0.251	180	0.001	0.821	229.690	0.006
Weighted median	0.632	0.433	0.923	0.018					
Inverse variance weighted	0.558	0.423	0.735	3.443E-05					
Simple mode	0.806	0.265	2.457	0.705					
Weighted mode	0.664	0.237	1.863	0.438					
**Intelligence**									
MR Egger	0.624	0.311	1.255	0.188	142	0.007	0.342	197.281	0.001
Weighted median	0.806	0.672	0.965	0.019					
Inverse variance weighted	0.871	0.760	0.999	0.048					
Simple mode	0.710	0.400	1.260	0.244					
Weighted mode	0.693	0.410	1.171	0.173					
**Income**									
MR Egger	1.108	0.302	4.068	0.878	45	-0.005	0.722	57.082	0.089
Weighted median	0.787	0.566	1.093	0.152					
Inverse variance weighted	0.877	0.682	1.129	0.309					
Simple mode	0.757	0.375	1.528	0.441					
Weighted mode	0.769	0.390	1.515	0.452					
**Social deprivation**									
MR Egger	7.034	0.269	183.613	0.258	18	-0.018	0.423	19.436	0.304
Weighted median	1.834	0.920	3.654	0.085					
Inverse variance weighted	1.821	1.075	3.085	0.026					
Simple mode	1.649	0.486	5.597	0.433					
Weighted mode	1.730	0.517	5.791	0.386					
**Attention-deficit hyperactivity disorder**									
MR Egger	0.857	0.571	1.285	0.480	9	0.020	0.316	6.816	0.557
Weighted median	1.056	0.919	1.213	0.446					
Inverse variance weighted	1.064	0.958	1.181	0.246					
Simple mode	1.091	0.879	1.355	0.452					
Weighted mode	1.021	0.839	1.242	0.844					
**Bipolar disorder**									
MR Egger	0.836	0.483	1.446	0.535	13	0.019	0.464	7.115	0.850
Weighted median	1.099	0.978	1.235	0.111					
Inverse variance weighted	1.030	0.942	1.127	0.512					
Simple mode	1.130	0.926	1.379	0.253					
Weighted mode	1.125	0.921	1.373	0.270					
**Major depressive disorder**									
MR Egger	1.220	0.575	2.591	0.608	33	-0.001	0.958	40.685	0.140
Weighted median	1.215	0.979	1.508	0.077					
Inverse variance weighted	1.196	1.015	1.409	0.032					
Simple mode	1.274	0.765	2.123	0.359					
Weighted mode	1.228	0.740	2.038	0.433					
**Schizophrenia**									
MR Egger	0.994	0.867	1.140	0.929	207	-2.271E-04	0.956	217.592	0.276
Weighted median	1.014	0.967	1.064	0.559					
Inverse variance weighted	0.990	0.957	1.025	0.568					
Simple mode	1.053	0.901	1.231	0.516					
Weighted mode	1.057	0.913	1.225	0.457					

OR, odds ratio; CI, confidence interval; SNPs, single-nucleotide polymorphisms

**Table 3:** Results of the MRPRESSO testing

Exposure	Outcome	Outliers	Global test	Outlier corrected	P_distortion_
Years of schooling	Sleep apnea	rs363096 rs62444881 rs12574281 rs11620355	1.000E-04	2.546E-06	0.677
College or University degree	Sleep apnea	rs11155821	0.006	1.075E-05	0.800
Intelligence	Sleep apnea	rs58593843	0.001	0.022	0.804
Income	Sleep apnea	NA	0.088	NA	NA
Social deprivation	Sleep apnea	NA	0.305	NA	NA
ADHD	Sleep apnea	NA	0.559	NA	NA
Bipolar disorder	Sleep apnea	NA	0.863	NA	NA
Major depressive disorder	Sleep apnea	NA	0.139	NA	NA
Schizophrenia	Sleep apnea	NA	0.284	NA	NA
Sleep apnea	Years of schooling	rs10928560 rs4837016 rs9937053	1.000E-04	0.057	1.000
Sleep apnea	College or university degree	rs4837016 rs9937053	0.003	0.485	0.669
Sleep apnea	Intelligence	1	0.033	0.149	0.490
Sleep apnea	Income	4	0.035	0.108	0.661
Sleep apnea	Social deprivation	1	0.038	0.352	0.618
Sleep apnea	ADHD	NA	0.707	NA	NA
Sleep apnea	Bipolar disorder	NA	0.929	NA	NA
Sleep apnea	Major depressive disorder	NA	NA	NA	NA
Sleep apnea	Schizophrenia	NA	0.726	NA	NA
Sleep apnea	Anxiety disorders	NA	0.189	NA	NA

ADHD, attention-deficit hyperactivity disorder; NA, not available

## The causal effect of SA on mental health and socioeconomic status

Significant heterogeneity was found by Cochrane’s Q test (Table 4, P < 0.05), and an IVW approach with the multiplicative random-effect model was applied to the main analyses. Reverse MR analysis failed to obtain a causal effect between genetic liability to SA with mental health and socioeconomic status using the IVW method (Table 4). No directional pleiotropy was found by the ME-Egger regression analysis (P > 0.05). However, MR-PRESSO detected outliers, revealing that the IVs of SA and outcome had a substantial horizontal pleiotropy (P < 0.05). Results did not change substantially after the removal of outliers (Table 3).

**Table 4:** Results of the MR study testing causing the association between sleep apnea and mental health and socioeconomic status

Analysis	OR	Lower 95%CI	Upper 95%CI	p	SNPs, n	Horizontal pleiotropy		Heterogeneity	
						Egger intercept	P	Q	P
**Years of schooling**									
MR Egger	0.866	0.605	1.238	0.487	5	0.014	0.453	39.029	6.871E-08
Weighted median	1.025	0.993	1.058	0.125					
Inverse variance weighted	1.010	0.943	1.081	0.786					
Simple mode	1.021	0.918	1.137	0.720					
Weighted mode	1.029	0.985	1.074	0.268					
**College or university degree**									
MR Egger	0.967	0.781	1.198	0.781	5	0.004	0.740	26.411	2.615E-05
Weighted median	1.020	0.999	1.041	0.060					
Inverse variance weighted	1.006	0.969	1.045	0.760					
Simple mode	1.026	0.990	1.065	0.235					
Weighted mode	1.026	1.003	1.049	0.093					
**Intelligence**									
MR Egger	0.853	0.608	1.197	0.426	5	0.016	0.382	15.143	0.004
Weighted median	1.055	1.010	1.102	0.017					
Inverse variance weighted	1.015	0.951	1.083	0.658					
Simple mode	1.048	0.991	1.108	0.178					
Weighted mode	1.050	1.006	1.096	0.089					
**Income**									
MR Egger	0.753	0.618	0.919	0.068	5	0.025	0.074	13.684	0.008
Weighted median	0.970	0.930	1.012	0.160					
Inverse variance weighted	0.984	0.924	1.048	0.613					
Simple mode	0.935	0.871	1.003	0.134					
Weighted mode	0.966	0.917	1.017	0.256					
**Social deprivation**									
MR Egger	1.186	0.977	1.441	0.184	5	-0.016	0.173	13.523	0.009
Weighted median	0.990	0.958	1.023	0.552					
Inverse variance weighted	0.998	0.951	1.046	0.919					
Simple mode	0.987	0.945	1.031	0.584					
Weighted mode	0.986	0.949	1.024	0.505					
**Attention-deficit hyperactivity disorder**									
MR Egger	1.856	0.648	5.315	0.368	4	-0.055	0.372	1.537	0.674
Weighted median	1.011	0.831	1.229	0.916					
Inverse variance weighted	1.015	0.854	1.205	0.868					
Simple mode	0.932	0.701	1.239	0.662					
Weighted mode	1.052	0.822	1.347	0.713					
**Bipolar disorder**									
MR Egger	0.988	0.422	2.311	0.979	5	0.005	0.906	0.672	0.955
Weighted median	1.058	0.863	1.296	0.590					
Inverse variance weighted	1.043	0.881	1.235	0.625					
Simple mode	0.956	0.734	1.246	0.757					
Weighted mode	1.094	0.874	1.369	0.479					
**Major depressive disorder**									
Inverse variance weighted	1.033	0.744	1.436	0.845	2	NA	NA	18.057	2.143E-05
**Schizophrenia**									
MR Egger	1.219	0.742	2.002	0.491	5	-1.839E-02	0.487	2.138	0.710
Weighted median	0.990	0.885	1.107	0.860					
Inverse variance weighted	1.001	0.914	1.097	0.975					
Simple mode	1.003	0.869	1.158	0.965					
Weighted mode	0.983	0.860	1.124	0.817					
**Anxiety disorders**									
MR Egger	0.999	0.986	1.013	0.932	5	1.197E-01	0.884	7.325	0.120
Weighted median	1.000	0.997	1.002	0.674					
Inverse variance weighted	1.000	0.998	1.003	0.714					
Simple mode	0.999	0.995	1.003	0.735					
Weighted mode	0.999	0.996	1.001	0.450					

OR, odds ratio; CI, confidence interval; SNPs, single-nucleotide polymorphisms

## Discussion

There is evidence that having a higher level of education protects against SA, whereas people with mental health conditions are more likely to develop SA [8, 9]. In our research, MR analysis was carried out to assess the potential causation between mental health, socioeconomic status, and SA, which employs random allocation of alleles to duplicate the randomized procedure in double-blind clinical trials. Appling large-scale summary statistics from mental health, socioeconomic status GWAS, and SA GWAS, we found that higher education attainment was strongly correlated with a lower risk of SA, and social deprivation as well as major depressive disorder were strongly associated with a higher risk of SA.

Under the assumption that the association of each genetic variant with the exposure is independent of the pleiotropic effect, the MR-Egger regression provides a valid effect estimate even when all of the genetic variants and invalid instruments are present [21]. The intercept of the MR-Egger regression was corrected to assess for pleiotropy bias. It is a reasonable assumption in this context because no pleiotropic effect of the variant was observed after a look-up of all SNPs and the MR-Egger intercept. The MR-Egger intercept results in our study indicated that there was no pleiotropic effect that affected the outcome through factors other than exposure. MR-PRESSO is used in multi-instrument MR analysis to detect and correct for horizontal pleiotropic outliers through outlier removal [22]. Some outliers were found in the study; however, after the outliers were removed, the results did not change significantly. A leave-one-out analysis was performed to estimate the potential influence of a single SNP, and the results showed that the association between mental health, socioeconomic status, and SA was not significantly driven by any individual SNP. The aforementioned results evaluate the robustness of the MR results.

There have been a few observational studies on the connection between education and SA [9], but due to factors including limited cohorts, measurement inaccuracy, and methodological restrictions, their findings are not reliable. Our result may provide some of the strongest evidence to evaluate the causal role of educational attainment in SA because the questions described previously can be partially or entirely avoided by a two-sample MR method. Our data improved statistical reliability by using summary statistics from the greatest GWAS studies for educational attainment and SA. Our results were in line with earlier MR research investigating the relationship between education level and SA [29].

A connection between low income and an increased risk for SA was shown by earlier observational research [10]. Our findings did not support a causal relationship between income and SA, which is inconsistent. This could be explained by the lower sensitivity and positive predictive value of the Beilin Questionnaire in detecting SA in the black community [10]. Additionally, observational findings supported the reported link between social deprivation and sleep disturbances [30], and the evidence from our study suggests that social deprivation is a risk factor that causes SA. There is little research on this relationship, thus further research is required.

It is significant to emphasize that our study did not find evidence for a generalized role for mental health problems in the risk of SA, but rather for a particular role for major depressive disorder. According to the prevalence meta-analysis, there may be a higher prevalence of SA in people with major depressive disorder, while there is insufficient data to suggest a higher incidence of SA in people with bipolar disorder and schizophrenia [31], which is consistent with our findings. Furthermore, our study could not support the contribution of SA to psychiatric disorders, including ADHD, bipolar disorder, major depressive disorder, schizophrenia, and anxiety disorders, while the available observational evidence was contradictory [5, 7, 32].

Our study possesses several important strengths, the most important of which is the MR design, which is appropriate for concluding causality. MR research might offer crucial insights into the relationships between mental health, socioeconomic position, and SA given the various difficulties in planning and executing RCTs in SA. Furthermore, the relationship between mental health and SA has never been studied in an MR setting.

However, it is crucial to notice that this MR study has several limitations. Firstly, the limited IV numbers weaken the proportion of phenotypic variance explained. As a result, the null findings for the relationship between anxiety disorders and SA do not necessarily indicate that anxiety disorders have no impact. Secondly, the phenotypes considered in this study rely on the definitions and samples utilized in the original GWASs, which are often highly heterogeneous in terms of the recruited population, the definition of the phenotype, and the assessment. Although the necessity for very large samples to uncover tiny genetic effects is what causes this heterogeneity, it might nonetheless have an impact on our conclusions. Thirdly, this study only includes people of European heritage due to data availability. Our findings may be limited in their applicability to other ethnicities. The uniformity of participants, on the other hand, ensures that there is little risk of confounding by population admixture. Fourthly, the lack of gender or age-specific GWAS summary statistics prevented us from examining potential gender and age variations in the link between mental health, socioeconomic position, and SA. Fifthly, the majority of the relationships that were identified only applied to adults, and they could vary throughout various developmental stages. Further MR analysis, after controlling for age, gender, and other environmental confounding factors, is required to reveal the causal relationship between mental health, socioeconomic status, and SA. Furthermore, results from other races may increase the study’s generalizability.

In conclusion, we adequately estimated the potential causal association between mental health, socioeconomic status, and SA. Education attainment was found to be associated with a lower risk of SA, and social deprivation and major depressive disorder were correlated with higher SA risk. Further study is needed to investigate the exact causal association and mechanism between mental health, socioeconomic status, and SA.

### Electronic supplementary material

Below is the link to the electronic supplementary material.

Supplementary Material 1

## Data Availability

The datasets analysed during the current study are available in the FinnGen database repository, https://www.finngen.fi/en/access_results [33].

## List of abbreviations

SA: sleep apnea
GWAS: genome-wide association studies
IV: instrumental variables
OR: odds ratio
CI: confidence interval
IVW: inverse variance weighted
MR analysis: Mendelian randomization analysis
MAF: minor allele frequency
SNPs: single-nucleotide polymorphisms
SSGAC: Social Science Genetic Association Consortium
UKB: UK Biobank
ADHD: attention-deficit hyperactivity disorder
PGC: Psychiatric Genomics Consortium
FINN: FinnGen research project
LD: linkage disequilibrium
LD: linkage disequilibrium
MR-PRESSO: MR Pleiotropy Residual Sum and Outlier
NA: not available
