# Supplementary material for: Assessing the causal associations of sleep apnea with mental health and socioeconomic status: a bidirectional two-sample Mendelian randomization
Source: BMC Med Genomics. 2024 Jan 22;17:27. doi: 10.1186/s12920-023-01783-6 (PMC10804749; doi:10.1186/s12920-023-01783-6)
Supplement: Supplementary file 1 — Supplementary Material 1 [file 12920_2023_1783_MOESM1_ESM.docx]

Supplementary Table 1 IVs of years of schooling.

|  | SNP | effect_allele | other_allele | se | beta | chr | pval | samplesize | F | |
| --- | --- | --- | --- | --- | --- | --- | --- | --- | --- | --- |
| 1 | rs34394051 | G | A | 0.002 | 0.014 | 1 | 6.200E-09 | 766345 | | 33.640 |
| 2 | rs12028010 | C | T | 0.002 | -0.017 | 1 | 4.510E-17 | 766345 | | 70.493 |
| 3 | rs1008078 | T | C | 0.002 | -0.017 | 1 | 1.200E-23 | 766345 | | 100.927 |
| 4 | rs12134151 | C | G | 0.002 | -0.012 | 1 | 2.420E-13 | 766345 | | 53.634 |
| 5 | rs10797055 | G | A | 0.002 | 0.010 | 1 | 1.040E-08 | 766345 | | 32.862 |
| 6 | rs2820314 | C | A | 0.002 | -0.011 | 1 | 9.340E-10 | 766345 | | 37.346 |
| 7 | rs4846724 | A | G | 0.002 | 0.010 | 1 | 2.260E-09 | 766345 | | 35.859 |
| 8 | rs59123361 | A | G | 0.003 | -0.021 | 1 | 5.869E-13 | 766345 | | 51.781 |
| 9 | rs4839155 | G | T | 0.002 | -0.013 | 1 | 3.940E-10 | 766345 | | 39.125 |
| 10 | rs10798418 | T | C | 0.002 | -0.010 | 1 | 3.290E-08 | 766345 | | 30.601 |
| 11 | rs3747631 | C | G | 0.002 | 0.022 | 1 | 2.970E-26 | 766345 | | 112.584 |
| 12 | rs622169 | T | C | 0.002 | 0.010 | 1 | 1.890E-08 | 766345 | | 31.499 |
| 13 | rs301800 | C | T | 0.002 | -0.015 | 1 | 1.330E-11 | 766345 | | 45.804 |
| 14 | rs74701752 | T | G | 0.003 | 0.016 | 1 | 2.380E-08 | 766345 | | 31.164 |
| 15 | rs1620977 | G | A | 0.002 | -0.020 | 1 | 1.140E-25 | 766345 | | 110.089 |
| 16 | rs1569092 | A | G | 0.002 | 0.018 | 1 | 1.160E-14 | 766345 | | 59.633 |
| 17 | rs10875121 | C | G | 0.002 | 0.018 | 1 | 5.530E-16 | 766345 | | 65.854 |
| 18 | rs575113 | A | G | 0.002 | 0.013 | 1 | 5.300E-12 | 766345 | | 47.729 |
| 19 | rs663234 | G | C | 0.002 | -0.010 | 1 | 7.390E-09 | 766345 | | 33.361 |
| 20 | rs2901616 | A | G | 0.002 | 0.009 | 1 | 3.770E-08 | 766345 | | 30.282 |
| 21 | rs79523955 | G | A | 0.003 | -0.018 | 1 | 1.870E-10 | 766345 | | 40.545 |
| 22 | rs2819336 | C | T | 0.002 | -0.018 | 1 | 5.460E-25 | 766345 | | 106.661 |
| 23 | rs9436866 | C | A | 0.003 | 0.019 | 1 | 7.451E-11 | 766345 | | 42.408 |
| 24 | rs3026996 | C | A | 0.002 | -0.015 | 1 | 1.050E-14 | 766345 | | 59.654 |
| 25 | rs35039375 | G | A | 0.003 | -0.020 | 1 | 1.220E-11 | 766345 | | 45.805 |
| 26 | rs16854920 | C | T | 0.002 | 0.010 | 1 | 2.510E-08 | 766345 | | 30.953 |
| 27 | rs71646142 | T | C | 0.002 | 0.013 | 1 | 3.110E-09 | 766345 | | 35.121 |
| 28 | rs3897821 | G | A | 0.002 | -0.015 | 1 | 8.251E-17 | 766345 | | 69.630 |
| 29 | rs72807818 | A | G | 0.003 | 0.019 | 2 | 2.790E-14 | 766345 | | 57.748 |
| 30 | rs1106090 | A | G | 0.002 | 0.012 | 2 | 2.090E-11 | 766345 | | 44.928 |
| 31 | rs9679654 | C | T | 0.002 | 0.010 | 2 | 1.290E-09 | 766345 | | 36.701 |
| 32 | rs1427298 | T | C | 0.002 | 0.010 | 2 | 3.280E-09 | 766345 | | 35.168 |
| 33 | rs13422673 | T | C | 0.002 | -0.012 | 2 | 1.740E-12 | 766345 | | 49.910 |
| 34 | rs11678980 | A | G | 0.002 | -0.017 | 2 | 4.290E-24 | 766345 | | 102.810 |
| 35 | rs62183776 | T | C | 0.002 | -0.013 | 2 | 1.650E-09 | 766345 | | 36.333 |
| 36 | rs4972400 | A | G | 0.002 | 0.012 | 2 | 1.700E-10 | 766345 | | 40.790 |
| 37 | rs11694904 | T | C | 0.002 | 0.012 | 2 | 4.780E-11 | 766345 | | 43.133 |
| 38 | rs4667025 | A | G | 0.002 | 0.010 | 2 | 3.850E-08 | 766345 | | 30.250 |
| 39 | rs1455350 | A | T | 0.002 | -0.016 | 2 | 2.610E-21 | 766345 | | 90.138 |
| 40 | rs62184480 | T | C | 0.002 | -0.015 | 2 | 1.280E-15 | 766345 | | 64.000 |
| 41 | rs112806496 | G | C | 0.003 | 0.019 | 2 | 8.280E-10 | 766345 | | 37.591 |
| 42 | rs7594904 | C | T | 0.002 | 0.010 | 2 | 2.050E-08 | 766345 | | 31.373 |
| 43 | rs13010566 | C | A | 0.002 | 0.011 | 2 | 4.590E-10 | 766345 | | 38.879 |
| 44 | rs10191758 | G | A | 0.002 | 0.016 | 2 | 9.601E-21 | 766345 | | 86.862 |
| 45 | rs7603132 | A | G | 0.002 | 0.013 | 2 | 9.170E-10 | 766345 | | 37.523 |
| 46 | rs11681861 | G | T | 0.003 | -0.014 | 2 | 2.880E-08 | 766345 | | 30.698 |
| 47 | rs16846463 | G | A | 0.003 | -0.023 | 2 | 1.380E-15 | 766345 | | 63.549 |
| 48 | rs67890737 | A | C | 0.002 | -0.011 | 2 | 2.010E-10 | 766345 | | 40.632 |
| 49 | rs76076331 | T | C | 0.002 | 0.019 | 2 | 4.400E-14 | 766345 | | 57.039 |
| 50 | rs10189857 | G | A | 0.002 | -0.017 | 2 | 6.700E-24 | 766345 | | 101.762 |
| 51 | rs6731373 | A | G | 0.002 | -0.013 | 2 | 3.470E-12 | 766345 | | 48.153 |
| 52 | rs62157915 | C | T | 0.003 | 0.021 | 2 | 1.960E-09 | 766345 | | 36.104 |
| 53 | rs10205801 | A | G | 0.002 | -0.011 | 2 | 7.170E-10 | 766345 | | 37.920 |
| 54 | rs6731967 | C | G | 0.002 | -0.012 | 2 | 2.360E-09 | 766345 | | 35.519 |
| 55 | rs10856785 | T | C | 0.002 | -0.011 | 2 | 3.830E-09 | 766345 | | 34.761 |
| 56 | rs12468040 | G | T | 0.002 | -0.014 | 2 | 2.460E-16 | 766345 | | 66.959 |
| 57 | rs11123818 | A | G | 0.002 | 0.021 | 2 | 1.720E-32 | 766345 | | 141.406 |
| 58 | rs2570497 | T | C | 0.002 | -0.012 | 2 | 3.030E-12 | 766345 | | 48.527 |
| 59 | rs1947114 | G | A | 0.002 | 0.011 | 2 | 2.640E-08 | 766345 | | 31.115 |
| 60 | rs1882273 | C | G | 0.002 | -0.012 | 2 | 1.090E-11 | 766345 | | 46.255 |
| 61 | rs13029509 | A | G | 0.002 | -0.010 | 2 | 7.170E-10 | 766345 | | 38.076 |
| 62 | rs77719387 | A | T | 0.007 | -0.046 | 3 | 2.460E-10 | 766345 | | 40.094 |
| 63 | rs9882532 | C | T | 0.002 | -0.012 | 3 | 8.170E-12 | 766345 | | 46.579 |
| 64 | rs13090388 | T | C | 0.002 | 0.029 | 3 | 4.290E-54 | 766345 | | 240.250 |
| 65 | rs6803651 | T | G | 0.002 | 0.011 | 3 | 4.360E-11 | 766345 | | 43.238 |
| 66 | rs9289300 | C | T | 0.002 | 0.015 | 3 | 1.100E-10 | 766345 | | 41.751 |
| 67 | rs7650602 | C | T | 0.002 | 0.009 | 3 | 4.110E-08 | 766345 | | 30.154 |
| 68 | rs35475880 | T | G | 0.002 | -0.015 | 3 | 3.800E-13 | 766345 | | 52.772 |
| 69 | rs55736314 | G | C | 0.002 | 0.014 | 3 | 1.630E-16 | 766345 | | 67.636 |
| 70 | rs66568921 | G | T | 0.002 | 0.016 | 3 | 7.490E-18 | 766345 | | 73.941 |
| 71 | rs79269403 | A | G | 0.002 | 0.014 | 3 | 1.170E-12 | 766345 | | 50.313 |
| 72 | rs6805241 | C | T | 0.002 | -0.014 | 3 | 3.090E-12 | 766345 | | 48.450 |
| 73 | rs17048855 | A | G | 0.002 | 0.012 | 3 | 3.270E-11 | 766345 | | 43.752 |
| 74 | rs4328757 | T | C | 0.002 | 0.011 | 3 | 9.390E-10 | 766345 | | 37.604 |
| 75 | rs115000530 | T | A | 0.004 | 0.029 | 3 | 3.300E-14 | 766345 | | 57.616 |
| 76 | rs2885198 | G | A | 0.002 | -0.010 | 3 | 1.810E-09 | 766345 | | 36.354 |
| 77 | rs73874335 | T | C | 0.004 | -0.020 | 3 | 3.400E-08 | 766345 | | 30.387 |
| 78 | rs77025239 | A | G | 0.002 | -0.014 | 3 | 1.330E-09 | 766345 | | 36.929 |
| 79 | rs363096 | C | T | 0.002 | 0.014 | 4 | 2.040E-15 | 766345 | | 62.796 |
| 80 | rs1450782 | G | T | 0.002 | -0.009 | 4 | 4.930E-08 | 766345 | | 29.838 |
| 81 | rs61747885 | T | G | 0.002 | 0.014 | 4 | 4.130E-09 | 766345 | | 34.634 |
| 82 | rs17598675 | C | T | 0.002 | 0.012 | 4 | 1.750E-12 | 766345 | | 49.744 |
| 83 | rs1949226 | A | T | 0.002 | -0.010 | 4 | 2.840E-08 | 766345 | | 30.758 |
| 84 | rs36083520 | C | T | 0.002 | 0.016 | 4 | 2.600E-13 | 766345 | | 53.362 |
| 85 | rs1595973 | T | C | 0.002 | -0.010 | 4 | 6.560E-09 | 766345 | | 33.546 |
| 86 | rs115454970 | T | G | 0.002 | -0.012 | 4 | 2.760E-09 | 766345 | | 35.459 |
| 87 | rs28373063 | C | G | 0.002 | 0.014 | 4 | 1.230E-09 | 766345 | | 36.790 |
| 88 | rs13130765 | C | G | 0.002 | -0.010 | 4 | 4.690E-09 | 766345 | | 34.355 |
| 89 | rs9995567 | A | G | 0.002 | 0.010 | 4 | 1.930E-08 | 766345 | | 31.436 |
| 90 | rs12503522 | T | C | 0.002 | -0.011 | 4 | 2.240E-09 | 766345 | | 35.809 |
| 91 | rs1391438 | C | T | 0.002 | -0.017 | 4 | 5.790E-20 | 766345 | | 83.278 |
| 92 | rs12643771 | T | C | 0.002 | 0.015 | 4 | 1.610E-16 | 766345 | | 68.063 |
| 93 | rs2347526 | C | T | 0.002 | 0.014 | 4 | 6.841E-15 | 766345 | | 60.735 |
| 94 | rs112687095 | A | G | 0.002 | 0.013 | 4 | 2.420E-08 | 766345 | | 30.994 |
| 95 | rs337637 | A | G | 0.002 | 0.011 | 4 | 2.110E-10 | 766345 | | 40.254 |
| 96 | rs13141210 | T | C | 0.002 | 0.014 | 4 | 2.260E-15 | 766345 | | 62.612 |
| 97 | rs13145650 | T | C | 0.003 | -0.019 | 4 | 3.800E-10 | 766345 | | 39.287 |
| 98 | rs969512 | T | A | 0.002 | 0.012 | 4 | 3.220E-12 | 766345 | | 48.688 |
| 99 | rs11732657 | A | G | 0.002 | -0.013 | 4 | 9.539E-11 | 766345 | | 41.822 |
| 100 | rs10940921 | G | T | 0.002 | -0.011 | 5 | 7.000E-10 | 766345 | | 37.854 |
| 101 | rs152603 | G | A | 0.002 | 0.010 | 5 | 9.470E-09 | 766345 | | 33.144 |
| 102 | rs10073890 | G | A | 0.002 | -0.013 | 5 | 1.110E-10 | 766345 | | 41.458 |
| 103 | rs892612 | C | A | 0.002 | 0.015 | 5 | 6.630E-10 | 766345 | | 38.158 |
| 104 | rs12519073 | T | C | 0.002 | -0.012 | 5 | 1.600E-09 | 766345 | | 36.537 |
| 105 | rs78721320 | A | G | 0.002 | 0.013 | 5 | 2.280E-09 | 766345 | | 35.617 |
| 106 | rs2441111 | A | G | 0.002 | 0.011 | 5 | 1.780E-10 | 766345 | | 40.885 |
| 107 | rs34316 | C | A | 0.002 | -0.020 | 5 | 3.350E-30 | 766345 | | 129.728 |
| 108 | rs17489649 | G | A | 0.002 | -0.014 | 5 | 1.570E-14 | 766345 | | 58.976 |
| 109 | rs702606 | C | T | 0.003 | -0.014 | 5 | 1.120E-08 | 766345 | | 32.581 |
| 110 | rs4700393 | G | A | 0.002 | 0.021 | 5 | 1.510E-34 | 766345 | | 150.567 |
| 111 | rs12332731 | A | T | 0.002 | 0.014 | 5 | 3.120E-10 | 766345 | | 39.725 |
| 112 | rs3890802 | A | G | 0.002 | -0.011 | 5 | 2.740E-09 | 766345 | | 35.188 |
| 113 | rs31940 | A | G | 0.002 | 0.015 | 5 | 3.240E-10 | 766345 | | 39.598 |
| 114 | rs17563464 | A | C | 0.002 | -0.015 | 5 | 2.890E-12 | 766345 | | 48.539 |
| 115 | rs1363862 | A | G | 0.002 | -0.012 | 5 | 1.020E-09 | 766345 | | 37.197 |
| 116 | rs1827540 | G | A | 0.002 | -0.011 | 5 | 4.500E-10 | 766345 | | 38.879 |
| 117 | rs6867851 | C | G | 0.002 | -0.012 | 5 | 3.970E-12 | 766345 | | 48.114 |
| 118 | rs74643044 | C | T | 0.004 | 0.023 | 5 | 1.800E-09 | 766345 | | 36.218 |
| 119 | rs1592757 | C | G | 0.002 | -0.010 | 5 | 5.890E-09 | 766345 | | 33.704 |
| 120 | rs406413 | T | A | 0.002 | -0.017 | 5 | 4.839E-16 | 766345 | | 65.773 |
| 121 | rs1584469 | T | C | 0.002 | -0.013 | 5 | 2.100E-12 | 766345 | | 49.607 |
| 122 | rs2545798 | A | T | 0.002 | -0.013 | 5 | 3.110E-15 | 766345 | | 61.958 |
| 123 | rs4352658 | T | C | 0.003 | -0.021 | 6 | 5.550E-12 | 766345 | | 47.377 |
| 124 | rs9320493 | G | A | 0.002 | -0.014 | 6 | 6.130E-09 | 766345 | | 33.737 |
| 125 | rs4392737 | G | A | 0.002 | -0.010 | 6 | 2.240E-08 | 766345 | | 31.438 |
| 126 | rs9503598 | A | G | 0.002 | 0.011 | 6 | 3.120E-10 | 766345 | | 39.815 |
| 127 | rs72828517 | C | T | 0.002 | 0.018 | 6 | 2.830E-16 | 766345 | | 67.181 |
| 128 | rs9342482 | T | G | 0.002 | 0.013 | 6 | 1.360E-10 | 766345 | | 41.168 |
| 129 | rs9386319 | G | A | 0.002 | 0.010 | 6 | 1.270E-08 | 766345 | | 32.438 |
| 130 | rs9372625 | A | G | 0.002 | 0.024 | 6 | 6.759E-42 | 766345 | | 183.325 |
| 131 | rs11752914 | C | T | 0.002 | -0.012 | 6 | 2.130E-08 | 766345 | | 31.277 |
| 132 | rs6938002 | A | G | 0.002 | -0.010 | 6 | 5.410E-09 | 766345 | | 33.949 |
| 133 | rs9384679 | T | C | 0.002 | -0.010 | 6 | 4.880E-08 | 766345 | | 29.690 |
| 134 | rs10456918 | C | A | 0.002 | 0.015 | 6 | 3.670E-11 | 766345 | | 43.950 |
| 135 | rs6557171 | C | T | 0.002 | 0.016 | 6 | 4.150E-18 | 766345 | | 74.952 |
| 136 | rs9349956 | C | A | 0.002 | 0.019 | 6 | 6.281E-17 | 766345 | | 69.890 |
| 137 | rs2182505 | C | T | 0.002 | -0.011 | 6 | 1.640E-08 | 766345 | | 31.993 |
| 138 | rs2179152 | C | T | 0.002 | 0.015 | 6 | 1.210E-16 | 766345 | | 68.344 |
| 139 | rs2256965 | G | A | 0.002 | -0.011 | 6 | 1.590E-10 | 766345 | | 41.076 |
| 140 | rs9386787 | G | A | 0.002 | 0.010 | 6 | 1.820E-08 | 766345 | | 31.757 |
| 141 | rs4870482 | G | C | 0.002 | -0.011 | 6 | 1.270E-08 | 766345 | | 32.490 |
| 142 | rs3800546 | G | C | 0.002 | -0.012 | 6 | 9.730E-10 | 766345 | | 37.185 |
| 143 | rs17551064 | G | A | 0.002 | -0.015 | 7 | 8.620E-11 | 766345 | | 42.137 |
| 144 | rs7803932 | A | G | 0.002 | 0.014 | 7 | 2.440E-10 | 766345 | | 40.036 |
| 145 | rs2283076 | G | A | 0.002 | -0.011 | 7 | 2.070E-08 | 766345 | | 31.393 |
| 146 | rs4726070 | A | G | 0.002 | 0.013 | 7 | 5.950E-13 | 766345 | | 51.691 |
| 147 | rs7808399 | G | A | 0.002 | 0.011 | 7 | 3.780E-10 | 766345 | | 39.154 |
| 148 | rs36119825 | A | G | 0.002 | 0.011 | 7 | 4.820E-10 | 766345 | | 38.643 |
| 149 | rs113615161 | T | C | 0.003 | -0.015 | 7 | 3.970E-09 | 766345 | | 34.669 |
| 150 | rs7796203 | A | G | 0.002 | -0.011 | 7 | 3.600E-10 | 766345 | | 39.447 |
| 151 | rs2971970 | G | T | 0.002 | 0.017 | 7 | 1.250E-15 | 766345 | | 63.846 |
| 152 | rs62444881 | T | C | 0.002 | 0.018 | 7 | 5.790E-17 | 766345 | | 69.957 |
| 153 | rs62439690 | A | G | 0.002 | -0.011 | 7 | 2.180E-08 | 766345 | | 31.395 |
| 154 | rs10240905 | C | T | 0.002 | 0.012 | 7 | 3.790E-11 | 766345 | | 43.471 |
| 155 | rs11772580 | T | G | 0.002 | -0.012 | 7 | 2.570E-09 | 766345 | | 35.583 |
| 156 | rs4073894 | A | G | 0.002 | 0.015 | 7 | 5.400E-13 | 766345 | | 52.168 |
| 157 | rs113520408 | A | G | 0.002 | 0.013 | 7 | 1.020E-11 | 766345 | | 46.127 |
| 158 | rs320693 | C | G | 0.002 | 0.012 | 7 | 1.580E-12 | 766345 | | 50.160 |
| 159 | rs10215082 | G | A | 0.002 | 0.013 | 7 | 3.330E-14 | 766345 | | 57.389 |
| 160 | rs535307 | G | A | 0.002 | -0.010 | 7 | 4.730E-08 | 766345 | | 29.774 |
| 161 | rs34853711 | C | G | 0.002 | -0.016 | 7 | 9.881E-15 | 766345 | | 60.025 |
| 162 | rs79265434 | G | A | 0.003 | 0.023 | 7 | 6.080E-19 | 766345 | | 79.156 |
| 163 | rs6959891 | G | A | 0.002 | -0.011 | 7 | 1.740E-09 | 766345 | | 36.127 |
| 164 | rs35417702 | T | C | 0.002 | -0.014 | 7 | 1.930E-17 | 766345 | | 72.250 |
| 165 | rs7016302 | G | C | 0.002 | 0.012 | 8 | 4.980E-08 | 766345 | | 29.722 |
| 166 | rs2923431 | C | G | 0.002 | 0.011 | 8 | 9.840E-11 | 766345 | | 41.955 |
| 167 | rs77702622 | A | G | 0.004 | -0.024 | 8 | 2.990E-12 | 766345 | | 48.602 |
| 168 | rs67885444 | T | C | 0.002 | 0.014 | 8 | 1.480E-09 | 766345 | | 36.728 |
| 169 | rs4733264 | C | G | 0.002 | -0.010 | 8 | 4.530E-08 | 766345 | | 30.061 |
| 170 | rs7833201 | C | G | 0.003 | -0.015 | 8 | 5.050E-09 | 766345 | | 34.191 |
| 171 | rs2725370 | C | T | 0.002 | 0.015 | 8 | 1.970E-16 | 766345 | | 67.468 |
| 172 | rs1866823 | A | G | 0.002 | 0.010 | 8 | 3.810E-09 | 766345 | | 34.817 |
| 173 | rs837080 | C | T | 0.002 | 0.011 | 8 | 1.430E-10 | 766345 | | 41.262 |
| 174 | rs59480703 | C | G | 0.002 | -0.012 | 8 | 8.320E-09 | 766345 | | 33.103 |
| 175 | rs7012546 | T | C | 0.002 | 0.010 | 8 | 4.930E-09 | 766345 | | 34.413 |
| 176 | rs2447535 | G | A | 0.002 | 0.012 | 8 | 1.690E-10 | 766345 | | 40.753 |
| 177 | rs1566085 | T | G | 0.002 | 0.016 | 8 | 6.901E-22 | 766345 | | 92.542 |
| 178 | rs113182709 | A | G | 0.006 | 0.032 | 8 | 1.290E-08 | 766345 | | 32.351 |
| 179 | rs7863447 | A | G | 0.002 | 0.017 | 9 | 5.910E-13 | 766345 | | 51.865 |
| 180 | rs17425572 | G | A | 0.002 | -0.012 | 9 | 6.890E-13 | 766345 | | 51.840 |
| 181 | rs111821073 | T | C | 0.002 | 0.014 | 9 | 4.850E-09 | 766345 | | 34.151 |
| 182 | rs10963297 | G | C | 0.002 | 0.019 | 9 | 7.360E-22 | 766345 | | 92.471 |
| 183 | rs7029718 | A | G | 0.002 | 0.024 | 9 | 1.850E-44 | 766345 | | 196.483 |
| 184 | rs1105307 | A | G | 0.002 | -0.012 | 9 | 1.670E-09 | 766345 | | 36.185 |
| 185 | rs7031698 | C | T | 0.002 | 0.012 | 9 | 1.260E-09 | 766345 | | 36.702 |
| 186 | rs4743923 | C | T | 0.002 | 0.010 | 9 | 2.420E-08 | 766345 | | 31.322 |
| 187 | rs1051474 | C | T | 0.002 | 0.013 | 9 | 4.860E-12 | 766345 | | 47.889 |
| 188 | rs4382592 | G | T | 0.002 | 0.016 | 9 | 1.010E-18 | 766345 | | 78.203 |
| 189 | rs10760023 | G | C | 0.002 | 0.011 | 9 | 2.120E-09 | 766345 | | 35.804 |
| 190 | rs12375949 | C | T | 0.002 | 0.014 | 9 | 3.310E-17 | 766345 | | 70.775 |
| 191 | rs12682775 | C | T | 0.002 | 0.012 | 9 | 5.990E-09 | 766345 | | 33.856 |
| 192 | rs7924036 | T | G | 0.002 | 0.015 | 10 | 1.070E-18 | 766345 | | 77.959 |
| 193 | rs17126938 | C | T | 0.003 | 0.015 | 10 | 8.140E-10 | 766345 | | 37.749 |
| 194 | rs1925576 | G | A | 0.002 | 0.010 | 10 | 4.940E-09 | 766345 | | 33.994 |
| 195 | rs73344830 | G | A | 0.002 | -0.017 | 10 | 1.950E-23 | 766345 | | 100.000 |
| 196 | rs1291818 | C | T | 0.002 | -0.011 | 10 | 1.780E-10 | 766345 | | 40.734 |
| 197 | rs10994777 | A | G | 0.002 | 0.015 | 10 | 3.360E-10 | 766345 | | 39.603 |
| 198 | rs790647 | A | C | 0.002 | -0.015 | 10 | 2.170E-13 | 766345 | | 53.826 |
| 199 | rs4384309 | A | G | 0.002 | 0.011 | 10 | 2.520E-10 | 766345 | | 40.160 |
| 200 | rs3013014 | A | G | 0.002 | -0.010 | 10 | 2.920E-09 | 766345 | | 35.444 |
| 201 | rs7920624 | T | A | 0.002 | -0.012 | 10 | 3.970E-12 | 766345 | | 48.262 |
| 202 | rs55771711 | C | G | 0.002 | 0.016 | 10 | 5.410E-15 | 766345 | | 61.060 |
| 203 | rs72840994 | G | T | 0.002 | 0.012 | 10 | 7.770E-09 | 766345 | | 33.329 |
| 204 | rs10887801 | T | G | 0.002 | 0.011 | 10 | 2.270E-10 | 766345 | | 40.408 |
| 205 | rs7928622 | T | A | 0.002 | 0.010 | 11 | 2.520E-08 | 766345 | | 31.199 |
| 206 | rs80171383 | A | G | 0.002 | 0.015 | 11 | 1.830E-09 | 766345 | | 36.199 |
| 207 | rs11601122 | G | A | 0.002 | -0.019 | 11 | 2.240E-17 | 766345 | | 71.660 |
| 208 | rs894067 | A | G | 0.002 | 0.010 | 11 | 2.740E-09 | 766345 | | 35.386 |
| 209 | rs4945424 | A | C | 0.002 | -0.010 | 11 | 6.940E-09 | 766345 | | 33.654 |
| 210 | rs12574281 | C | A | 0.002 | 0.011 | 11 | 8.850E-10 | 766345 | | 37.446 |
| 211 | rs7481514 | G | A | 0.002 | 0.011 | 11 | 1.560E-09 | 766345 | | 36.270 |
| 212 | rs795230 | T | C | 0.002 | 0.010 | 11 | 2.970E-08 | 766345 | | 30.635 |
| 213 | rs77128898 | T | C | 0.005 | -0.028 | 11 | 9.470E-09 | 766345 | | 33.003 |
| 214 | rs76878669 | G | C | 0.002 | -0.014 | 11 | 8.670E-12 | 766345 | | 46.572 |
| 215 | rs510706 | C | G | 0.002 | 0.011 | 11 | 2.730E-09 | 766345 | | 35.403 |
| 216 | rs17565975 | A | G | 0.002 | -0.011 | 11 | 2.560E-11 | 766345 | | 44.601 |
| 217 | rs12804787 | G | A | 0.003 | -0.018 | 11 | 2.960E-08 | 766345 | | 30.774 |
| 218 | rs4757957 | C | G | 0.002 | 0.014 | 11 | 1.810E-14 | 766345 | | 58.722 |
| 219 | rs11023749 | A | G | 0.002 | 0.011 | 11 | 2.960E-10 | 766345 | | 39.550 |
| 220 | rs9704097 | A | C | 0.002 | -0.010 | 11 | 1.610E-09 | 766345 | | 36.281 |
| 221 | rs10765775 | A | G | 0.002 | 0.015 | 11 | 2.620E-17 | 766345 | | 71.479 |
| 222 | rs1143770 | T | C | 0.002 | 0.011 | 11 | 4.310E-11 | 766345 | | 43.621 |
| 223 | rs35309068 | G | T | 0.002 | 0.013 | 12 | 1.150E-14 | 766345 | | 59.678 |
| 224 | rs710629 | A | G | 0.002 | 0.011 | 12 | 2.960E-09 | 766345 | | 35.392 |
| 225 | rs7315713 | T | A | 0.002 | -0.010 | 12 | 4.340E-08 | 766345 | | 29.869 |
| 226 | rs401687 | C | G | 0.002 | 0.011 | 12 | 1.860E-11 | 766345 | | 45.285 |
| 227 | rs1558727 | T | C | 0.002 | -0.011 | 12 | 3.090E-10 | 766345 | | 39.542 |
| 228 | rs10773002 | T | A | 0.002 | -0.022 | 12 | 8.680E-29 | 766345 | | 123.695 |
| 229 | rs4766424 | G | C | 0.003 | -0.014 | 12 | 4.430E-08 | 766345 | | 29.867 |
| 230 | rs73301698 | A | G | 0.002 | -0.013 | 12 | 5.810E-10 | 766345 | | 38.524 |
| 231 | rs77835879 | G | A | 0.003 | -0.016 | 12 | 2.680E-08 | 766345 | | 30.903 |
| 232 | rs17110109 | C | T | 0.002 | 0.010 | 12 | 4.710E-09 | 766345 | | 34.172 |
| 233 | rs10862376 | A | T | 0.002 | 0.016 | 12 | 1.400E-11 | 766345 | | 45.718 |
| 234 | rs1671770 | C | A | 0.002 | -0.013 | 12 | 1.910E-09 | 766345 | | 36.216 |
| 235 | rs10772644 | C | G | 0.003 | 0.016 | 12 | 1.500E-09 | 766345 | | 36.541 |
| 236 | rs1689510 | C | G | 0.002 | 0.018 | 12 | 1.400E-22 | 766345 | | 95.714 |
| 237 | rs4964046 | G | A | 0.002 | 0.011 | 12 | 3.360E-09 | 766345 | | 34.996 |
| 238 | rs7977614 | G | A | 0.002 | 0.013 | 12 | 2.090E-11 | 766345 | | 44.782 |
| 239 | rs7993663 | C | T | 0.002 | 0.012 | 13 | 3.250E-11 | 766345 | | 43.946 |
| 240 | rs4497562 | G | A | 0.002 | -0.012 | 13 | 3.730E-10 | 766345 | | 39.323 |
| 241 | rs7321274 | G | A | 0.002 | -0.013 | 13 | 1.590E-09 | 766345 | | 36.514 |
| 242 | rs11620355 | A | G | 0.003 | 0.018 | 13 | 4.770E-09 | 766345 | | 34.262 |
| 243 | rs9529119 | G | C | 0.002 | -0.013 | 13 | 2.130E-10 | 766345 | | 40.298 |
| 244 | rs7332724 | T | C | 0.002 | -0.011 | 13 | 1.260E-09 | 766345 | | 36.959 |
| 245 | rs277828 | A | C | 0.002 | -0.011 | 13 | 2.710E-08 | 766345 | | 30.984 |
| 246 | rs2478208 | C | G | 0.002 | -0.011 | 13 | 4.820E-10 | 766345 | | 38.879 |
| 247 | rs1334297 | A | G | 0.002 | 0.024 | 13 | 3.060E-37 | 766345 | | 162.695 |
| 248 | rs9556958 | T | C | 0.002 | -0.011 | 13 | 2.380E-10 | 766345 | | 40.360 |
| 249 | rs2067854 | A | G | 0.002 | 0.015 | 14 | 1.380E-12 | 766345 | | 49.942 |
| 250 | rs8020034 | A | G | 0.002 | 0.018 | 14 | 1.170E-15 | 766345 | | 63.857 |
| 251 | rs11627087 | G | A | 0.003 | -0.018 | 14 | 3.710E-08 | 766345 | | 30.267 |
| 252 | rs4904523 | A | G | 0.002 | -0.009 | 14 | 3.710E-08 | 766345 | | 30.315 |
| 253 | rs736282 | C | T | 0.002 | -0.011 | 14 | 2.070E-10 | 766345 | | 40.509 |
| 254 | rs242093 | A | G | 0.002 | -0.010 | 14 | 2.070E-09 | 766345 | | 35.930 |
| 255 | rs8008382 | C | T | 0.002 | 0.012 | 14 | 6.121E-11 | 766345 | | 42.637 |
| 256 | rs176218 | T | G | 0.002 | 0.019 | 14 | 1.850E-18 | 766345 | | 76.705 |
| 257 | rs4442732 | G | A | 0.002 | -0.011 | 14 | 1.490E-09 | 766345 | | 36.479 |
| 258 | rs730384 | A | G | 0.002 | 0.010 | 14 | 3.010E-09 | 766345 | | 35.302 |
| 259 | rs2998315 | G | A | 0.002 | 0.013 | 14 | 1.120E-13 | 766345 | | 55.072 |
| 260 | rs2787101 | T | C | 0.002 | 0.010 | 14 | 2.500E-08 | 766345 | | 30.949 |
| 261 | rs56391344 | A | G | 0.002 | 0.016 | 15 | 1.340E-15 | 766345 | | 63.595 |
| 262 | rs4778058 | C | T | 0.002 | 0.010 | 15 | 2.400E-09 | 766345 | | 35.789 |
| 263 | rs117799466 | C | G | 0.002 | 0.012 | 15 | 2.910E-09 | 766345 | | 35.097 |
| 264 | rs2414072 | A | T | 0.002 | -0.010 | 15 | 4.350E-09 | 766345 | | 34.541 |
| 265 | rs28513670 | G | A | 0.002 | 0.015 | 15 | 5.061E-11 | 766345 | | 43.092 |
| 266 | rs4984541 | G | A | 0.002 | 0.012 | 15 | 2.770E-09 | 766345 | | 35.480 |
| 267 | rs11635092 | A | G | 0.002 | -0.012 | 15 | 3.890E-12 | 766345 | | 48.369 |
| 268 | rs6493265 | T | C | 0.002 | -0.014 | 15 | 1.700E-15 | 766345 | | 63.358 |
| 269 | rs2052285 | A | G | 0.002 | 0.011 | 16 | 1.340E-10 | 766345 | | 41.180 |
| 270 | rs3809634 | G | A | 0.002 | 0.011 | 16 | 1.090E-08 | 766345 | | 32.706 |
| 271 | rs60483752 | C | G | 0.002 | 0.011 | 16 | 3.890E-10 | 766345 | | 39.281 |
| 272 | rs117468730 | A | G | 0.006 | -0.035 | 16 | 3.790E-09 | 766345 | | 34.784 |
| 273 | rs9936270 | T | C | 0.002 | -0.014 | 16 | 6.430E-12 | 766345 | | 47.179 |
| 274 | rs35316276 | T | C | 0.002 | 0.012 | 16 | 1.520E-09 | 766345 | | 36.559 |
| 275 | rs9938678 | T | A | 0.002 | 0.014 | 16 | 4.120E-11 | 766345 | | 43.689 |
| 276 | rs4888746 | G | A | 0.002 | -0.010 | 16 | 4.150E-08 | 766345 | | 29.935 |
| 277 | rs34485537 | T | C | 0.002 | 0.011 | 16 | 5.670E-10 | 766345 | | 38.612 |
| 278 | rs9933256 | G | A | 0.002 | -0.011 | 16 | 4.570E-11 | 766345 | | 43.468 |
| 279 | rs4787457 | G | A | 0.002 | -0.017 | 16 | 3.730E-23 | 766345 | | 97.853 |
| 280 | rs818415 | G | T | 0.002 | 0.012 | 16 | 1.720E-08 | 766345 | | 31.801 |
| 281 | rs9914918 | A | G | 0.002 | 0.012 | 17 | 8.900E-10 | 766345 | | 37.346 |
| 282 | rs1381247 | C | T | 0.002 | -0.010 | 17 | 2.460E-08 | 766345 | | 30.980 |
| 283 | rs2302761 | T | C | 0.002 | 0.014 | 17 | 1.000E-10 | 766345 | | 41.971 |
| 284 | rs12602286 | T | G | 0.003 | 0.017 | 17 | 2.370E-11 | 766345 | | 44.497 |
| 285 | rs12940014 | C | T | 0.002 | 0.009 | 17 | 3.730E-08 | 766345 | | 30.315 |
| 286 | rs225291 | G | A | 0.002 | -0.012 | 17 | 1.840E-08 | 766345 | | 31.706 |
| 287 | rs11871429 | G | A | 0.002 | -0.014 | 17 | 1.920E-12 | 766345 | | 49.765 |
| 288 | rs74998289 | G | T | 0.002 | -0.018 | 17 | 1.310E-17 | 766345 | | 73.090 |
| 289 | rs11657342 | A | G | 0.002 | 0.014 | 17 | 1.940E-13 | 766345 | | 54.034 |
| 290 | rs613872 | T | G | 0.002 | -0.018 | 18 | 1.200E-14 | 766345 | | 59.433 |
| 291 | rs11081529 | C | T | 0.002 | -0.013 | 18 | 1.820E-12 | 766345 | | 49.680 |
| 292 | rs12955211 | A | T | 0.002 | 0.011 | 18 | 1.590E-09 | 766345 | | 36.330 |
| 293 | rs11663602 | A | C | 0.002 | -0.012 | 18 | 1.640E-10 | 766345 | | 40.758 |
| 294 | rs10460095 | A | G | 0.002 | -0.011 | 18 | 4.870E-10 | 766345 | | 38.862 |
| 295 | rs9964724 | T | C | 0.002 | 0.020 | 18 | 2.660E-27 | 766345 | | 116.829 |
| 296 | rs7233920 | A | G | 0.002 | -0.013 | 18 | 7.130E-11 | 766345 | | 42.379 |
| 297 | rs1618725 | T | C | 0.002 | 0.015 | 18 | 2.220E-17 | 766345 | | 72.055 |
| 298 | rs62097985 | T | C | 0.002 | -0.013 | 18 | 6.060E-14 | 766345 | | 56.076 |
| 299 | rs2554835 | A | G | 0.002 | 0.010 | 18 | 2.690E-08 | 766345 | | 30.977 |
| 300 | rs192436652 | T | C | 0.005 | -0.035 | 19 | 1.350E-10 | 766345 | | 41.172 |
| 301 | rs2905426 | T | G | 0.002 | 0.010 | 19 | 9.260E-09 | 766345 | | 32.825 |
| 302 | rs76608582 | A | C | 0.004 | 0.028 | 19 | 3.110E-10 | 766345 | | 39.534 |
| 303 | rs2287838 | A | G | 0.002 | -0.012 | 19 | 1.530E-11 | 766345 | | 45.385 |
| 304 | rs7257460 | C | T | 0.002 | -0.011 | 19 | 1.250E-09 | 766345 | | 36.702 |
| 305 | rs175325 | A | T | 0.002 | -0.012 | 20 | 1.110E-11 | 766345 | | 45.912 |
| 306 | rs4810227 | A | G | 0.002 | 0.013 | 20 | 3.570E-13 | 766345 | | 52.832 |
| 307 | rs4369924 | A | G | 0.002 | 0.014 | 20 | 5.820E-09 | 766345 | | 33.878 |
| 308 | rs6513959 | G | A | 0.002 | -0.012 | 20 | 1.880E-10 | 766345 | | 40.477 |
| 309 | rs16995054 | T | C | 0.002 | -0.014 | 20 | 2.520E-11 | 766345 | | 44.658 |
| 310 | rs6123924 | G | A | 0.002 | -0.015 | 20 | 7.549E-11 | 766345 | | 42.278 |
| 311 | rs6122735 | T | C | 0.002 | 0.011 | 20 | 1.490E-09 | 766345 | | 36.415 |
| 312 | rs7278859 | T | A | 0.002 | 0.010 | 21 | 4.150E-08 | 766345 | | 29.983 |
| 313 | rs1964927 | G | A | 0.002 | -0.014 | 21 | 9.899E-16 | 766345 | | 64.634 |
| 314 | rs743316 | C | T | 0.002 | -0.012 | 21 | 1.200E-08 | 766345 | | 32.457 |
| 315 | rs35532491 | T | A | 0.003 | 0.020 | 22 | 2.420E-12 | 766345 | | 49.245 |
| 316 | rs3788556 | C | T | 0.002 | -0.011 | 22 | 2.780E-11 | 766345 | | 44.289 |
| 317 | rs9616906 | A | G | 0.002 | 0.015 | 22 | 2.920E-18 | 766345 | | 75.751 |

Supplementary Table 2 IVs of college or university degree.

|  | SNP | effect_allele | other_allele | se | beta | chr | pval | samplesize | | F |
| --- | --- | --- | --- | --- | --- | --- | --- | --- | --- | --- |
| 1 | rs12028010 | C | T | 0.001 | -0.009 | 1 | 1.881E-11 | 334070 | | 45.094 |
| 2 | rs549845 | A | G | 0.001 | 0.012 | 1 | 1.372E-21 | 334070 | | 91.103 |
| 3 | rs11209952 | T | C | 0.001 | 0.010 | 1 | 1.818E-16 | 334070 | | 67.798 |
| 4 | rs59123361 | A | G | 0.002 | -0.012 | 1 | 1.076E-10 | 334070 | | 41.681 |
| 5 | rs9324380 | C | G | 0.002 | 0.012 | 1 | 6.885E-12 | 334070 | | 47.063 |
| 6 | rs1846228 | A | C | 0.001 | -0.007 | 1 | 1.277E-09 | 334070 | | 36.850 |
| 7 | rs7526112 | G | T | 0.001 | -0.010 | 1 | 2.850E-18 | 334070 | | 75.999 |
| 8 | rs680767 | G | A | 0.001 | -0.007 | 1 | 2.284E-08 | 334070 | | 31.238 |
| 9 | rs6673646 | C | T | 0.001 | -0.006 | 1 | 1.959E-08 | 334070 | | 31.536 |
| 10 | rs3943093 | T | C | 0.001 | -0.009 | 1 | 3.206E-12 | 334070 | | 48.562 |
| 11 | rs12735232 | C | T | 0.002 | 0.011 | 1 | 3.044E-12 | 334070 | | 48.664 |
| 12 | rs6676960 | T | C | 0.002 | 0.010 | 1 | 3.425E-08 | 334070 | | 30.452 |
| 13 | rs1325604 | A | G | 0.001 | -0.009 | 1 | 5.045E-10 | 334070 | | 38.662 |
| 14 | rs10801826 | T | C | 0.001 | -0.011 | 1 | 2.803E-19 | 334070 | | 80.582 |
| 15 | rs3747631 | C | G | 0.001 | 0.010 | 1 | 1.118E-13 | 334070 | | 55.154 |
| 16 | rs62172156 | C | T | 0.001 | 0.009 | 2 | 3.823E-14 | 334070 | | 57.262 |
| 17 | rs6704703 | C | T | 0.002 | 0.012 | 2 | 2.167E-08 | 334070 | | 31.341 |
| 18 | rs6429911 | C | T | 0.002 | 0.012 | 2 | 5.959E-09 | 334070 | | 33.850 |
| 19 | rs13397208 | A | C | 0.001 | 0.007 | 2 | 4.368E-08 | 334070 | | 29.980 |
| 20 | rs6735842 | C | T | 0.001 | 0.006 | 2 | 4.474E-08 | 334070 | | 29.934 |
| 21 | rs17235639 | G | A | 0.002 | -0.013 | 2 | 1.348E-08 | 334070 | | 32.262 |
| 22 | rs13033324 | G | A | 0.001 | 0.007 | 2 | 6.727E-10 | 334070 | | 38.101 |
| 23 | rs10189857 | G | A | 0.001 | -0.010 | 2 | 5.723E-17 | 334070 | | 70.078 |
| 24 | rs13018621 | T | A | 0.003 | -0.020 | 2 | 1.328E-09 | 334070 | | 36.774 |
| 25 | rs12619354 | C | T | 0.001 | -0.008 | 2 | 1.782E-10 | 334070 | | 40.695 |
| 26 | rs35811586 | T | C | 0.002 | 0.016 | 2 | 9.759E-11 | 334070 | | 41.872 |
| 27 | rs7577926 | T | C | 0.001 | 0.008 | 2 | 4.089E-09 | 334070 | | 34.583 |
| 28 | rs17328524 | T | C | 0.001 | -0.007 | 2 | 8.749E-09 | 334070 | | 33.103 |
| 29 | rs1455349 | G | A | 0.001 | -0.006 | 2 | 1.908E-08 | 334070 | | 31.588 |
| 30 | rs76076331 | T | C | 0.002 | 0.010 | 2 | 1.164E-08 | 334070 | | 32.548 |
| 31 | rs114460989 | T | G | 0.003 | -0.017 | 2 | 2.385E-08 | 334070 | | 31.154 |
| 32 | rs13426183 | A | G | 0.001 | 0.008 | 2 | 1.643E-09 | 334070 | | 36.360 |
| 33 | rs12991254 | C | T | 0.001 | 0.011 | 2 | 1.015E-20 | 334070 | | 87.143 |
| 34 | rs6707062 | C | T | 0.001 | 0.007 | 2 | 1.614E-08 | 334070 | | 31.913 |
| 35 | rs11678980 | A | G | 0.001 | -0.010 | 2 | 1.427E-16 | 334070 | | 68.276 |
| 36 | rs7582977 | C | T | 0.002 | -0.011 | 2 | 3.284E-08 | 334070 | | 30.534 |
| 37 | rs62182993 | A | G | 0.001 | -0.009 | 2 | 1.025E-13 | 334070 | | 55.323 |
| 38 | rs7561798 | G | A | 0.001 | -0.007 | 2 | 2.718E-10 | 334070 | | 39.870 |
| 39 | rs62246015 | T | C | 0.001 | 0.010 | 3 | 1.465E-15 | 334070 | | 63.684 |
| 40 | rs9826269 | C | A | 0.001 | -0.007 | 3 | 8.613E-09 | 334070 | 33.133 | |
| 41 | rs10934957 | C | G | 0.001 | 0.007 | 3 | 8.995E-09 | 334070 | | 33.049 |
| 42 | rs2061245 | A | G | 0.001 | -0.007 | 3 | 1.018E-08 | 334070 | | 32.808 |
| 43 | rs12637456 | A | T | 0.001 | -0.007 | 3 | 1.920E-08 | 334070 | | 31.575 |
| 44 | rs2271386 | A | G | 0.002 | 0.009 | 3 | 1.532E-08 | 334070 | | 32.014 |
| 45 | rs1873626 | A | T | 0.001 | -0.007 | 3 | 2.020E-09 | 334070 | | 35.957 |
| 46 | rs11915747 | G | C | 0.001 | 0.010 | 3 | 8.716E-18 | 334070 | | 73.792 |
| 47 | rs16828793 | G | T | 0.001 | -0.008 | 3 | 1.436E-08 | 334070 | | 32.140 |
| 48 | rs2341336 | T | C | 0.002 | -0.009 | 3 | 2.549E-08 | 334070 | | 31.025 |
| 49 | rs7613360 | T | C | 0.001 | -0.014 | 3 | 4.173E-35 | 334070 | | 152.867 |
| 50 | rs2885198 | G | A | 0.001 | -0.007 | 3 | 2.236E-09 | 334070 | | 35.758 |
| 51 | rs11711894 | T | C | 0.001 | 0.008 | 3 | 3.804E-09 | 334070 | | 34.724 |
| 52 | rs28624826 | T | C | 0.002 | 0.014 | 4 | 2.568E-08 | 334070 | | 31.011 |
| 53 | rs6839304 | G | A | 0.001 | 0.008 | 4 | 2.988E-10 | 334070 | | 39.685 |
| 54 | rs969512 | T | A | 0.001 | 0.008 | 4 | 4.624E-12 | 334070 | | 47.844 |
| 55 | rs10518019 | G | A | 0.001 | 0.008 | 4 | 2.798E-12 | 334070 | | 48.829 |
| 56 | rs2109661 | T | C | 0.001 | -0.007 | 4 | 3.738E-08 | 334070 | | 30.282 |
| 57 | rs35518360 | T | A | 0.002 | -0.012 | 4 | 9.481E-09 | 334070 | | 32.947 |
| 58 | rs4691576 | G | T | 0.001 | 0.008 | 4 | 5.922E-12 | 334070 | | 47.358 |
| 59 | rs2995803 | T | C | 0.001 | 0.009 | 4 | 4.694E-14 | 334070 | | 56.860 |
| 60 | rs2858088 | G | A | 0.001 | 0.007 | 4 | 1.447E-08 | 334070 | | 32.125 |
| 61 | rs1919489 | A | G | 0.001 | -0.008 | 4 | 1.439E-08 | 334070 | | 32.136 |
| 62 | rs34811474 | A | G | 0.001 | 0.008 | 4 | 3.073E-09 | 334070 | | 35.139 |
| 63 | rs1391441 | A | G | 0.001 | -0.010 | 4 | 1.567E-15 | 334070 | | 63.552 |
| 64 | rs360947 | A | G | 0.001 | 0.007 | 4 | 2.627E-10 | 334070 | | 39.936 |
| 65 | rs997779 | C | A | 0.001 | -0.008 | 4 | 1.428E-09 | 334070 | | 36.632 |
| 66 | rs10942580 | T | C | 0.002 | 0.010 | 5 | 1.305E-09 | 334070 | | 36.807 |
| 67 | rs6556982 | G | T | 0.001 | 0.006 | 5 | 3.276E-08 | 334070 | | 30.538 |
| 68 | rs3797699 | G | C | 0.001 | -0.007 | 5 | 2.408E-08 | 334070 | | 31.136 |
| 69 | rs1812587 | T | G | 0.001 | -0.007 | 5 | 2.554E-10 | 334070 | | 39.992 |
| 70 | rs12523278 | G | A | 0.001 | 0.010 | 5 | 5.466E-19 | 334070 | | 79.262 |
| 71 | rs640177 | A | T | 0.001 | -0.009 | 5 | 7.115E-16 | 334070 | | 65.108 |
| 72 | rs860815 | A | G | 0.001 | -0.009 | 5 | 2.919E-11 | 334070 | | 44.233 |
| 73 | rs4540171 | G | C | 0.001 | -0.007 | 5 | 1.036E-08 | 334070 | | 32.774 |
| 74 | rs42210 | C | G | 0.001 | -0.007 | 5 | 1.878E-08 | 334070 | | 31.619 |
| 75 | rs77451029 | C | T | 0.003 | 0.015 | 5 | 4.197E-09 | 334070 | | 34.532 |
| 76 | rs2545795 | C | A | 0.001 | -0.007 | 5 | 1.458E-08 | 334070 | | 32.110 |
| 77 | rs17563464 | A | C | 0.001 | -0.009 | 5 | 8.754E-12 | 334070 | | 46.593 |
| 78 | rs72829857 | G | A | 0.001 | 0.010 | 6 | 8.043E-13 | 334070 | | 51.276 |
| 79 | rs9357004 | C | T | 0.001 | 0.010 | 6 | 1.347E-17 | 334070 | | 72.932 |
| 80 | rs1007274 | T | C | 0.001 | -0.008 | 6 | 4.840E-08 | 334070 | | 29.781 |
| 81 | rs11155821 | C | T | 0.001 | 0.008 | 6 | 7.342E-12 | 334070 | | 46.937 |
| 82 | rs7757476 | A | G | 0.001 | 0.010 | 6 | 7.965E-11 | 334070 | | 42.269 |
| 83 | rs12203182 | A | G | 0.001 | 0.008 | 6 | 7.188E-09 | 334070 | | 33.485 |
| 84 | rs9401593 | C | A | 0.001 | 0.014 | 6 | 3.618E-36 | 334070 | | 157.728 |
| 85 | rs1536057 | T | C | 0.001 | -0.007 | 6 | 1.314E-08 | 334070 | | 32.312 |
| 86 | rs2269423 | C | A | 0.001 | -0.008 | 6 | 1.083E-10 | 334070 | | 41.669 |
| 87 | rs12526814 | C | T | 0.001 | 0.007 | 6 | 3.657E-09 | 334070 | | 34.800 |
| 88 | rs2268991 | T | C | 0.002 | -0.013 | 6 | 2.643E-08 | 334070 | | 30.955 |
| 89 | rs72979810 | G | C | 0.002 | -0.014 | 6 | 9.195E-10 | 334070 | | 37.491 |
| 90 | rs38857 | C | T | 0.001 | 0.007 | 7 | 3.419E-08 | 334070 | | 30.455 |
| 91 | rs4731366 | T | C | 0.001 | -0.007 | 7 | 1.130E-08 | 334070 | | 32.606 |
| 92 | rs10950862 | G | T | 0.001 | -0.008 | 7 | 4.714E-10 | 334070 | | 38.795 |
| 93 | rs11771370 | T | C | 0.002 | -0.009 | 7 | 6.757E-10 | 334070 | | 38.092 |
| 94 | rs2661876 | C | A | 0.001 | 0.007 | 7 | 1.353E-08 | 334070 | | 32.255 |
| 95 | rs11772108 | T | A | 0.001 | 0.008 | 7 | 2.789E-11 | 334070 | | 44.323 |
| 96 | rs4726070 | A | G | 0.001 | 0.007 | 7 | 8.582E-09 | 334070 | | 33.140 |
| 97 | rs1914391 | T | C | 0.001 | -0.008 | 7 | 4.000E-11 | 334070 | | 43.616 |
| 98 | rs11514731 | G | C | 0.001 | 0.011 | 7 | 4.932E-13 | 334070 | | 52.236 |
| 99 | rs1612548 | A | G | 0.001 | -0.009 | 7 | 3.488E-10 | 334070 | | 39.382 |
| 100 | rs2470964 | G | C | 0.001 | -0.008 | 7 | 6.747E-10 | 334070 | | 38.095 |
| 101 | rs7779631 | C | G | 0.001 | -0.011 | 7 | 1.941E-14 | 334070 | | 58.597 |
| 102 | rs62507575 | T | C | 0.001 | 0.006 | 8 | 2.725E-08 | 334070 | | 30.895 |
| 103 | rs4960987 | T | C | 0.001 | 0.008 | 8 | 2.692E-11 | 334070 | | 44.392 |
| 104 | rs10088132 | T | C | 0.001 | -0.008 | 8 | 1.776E-13 | 334070 | | 54.243 |
| 105 | rs2725377 | A | G | 0.001 | 0.007 | 8 | 7.477E-10 | 334070 | | 37.895 |
| 106 | rs2447535 | G | A | 0.001 | 0.007 | 8 | 2.450E-09 | 334070 | | 35.580 |
| 107 | rs11773992 | C | G | 0.002 | -0.008 | 8 | 3.619E-08 | 334070 | | 30.345 |
| 108 | rs2670012 | T | C | 0.001 | 0.007 | 8 | 2.369E-09 | 334070 | | 35.646 |
| 109 | rs56099375 | T | C | 0.001 | 0.007 | 8 | 3.929E-08 | 334070 | | 30.185 |
| 110 | rs118083122 | C | G | 0.005 | 0.028 | 8 | 2.382E-09 | 334070 | | 35.635 |
| 111 | rs35617898 | T | C | 0.001 | 0.008 | 9 | 1.734E-10 | 334070 | | 40.748 |
| 112 | rs6559365 | A | G | 0.001 | -0.007 | 9 | 1.629E-09 | 334070 | | 36.376 |
| 113 | rs12375949 | C | T | 0.001 | 0.008 | 9 | 5.514E-13 | 334070 | | 52.017 |
| 114 | rs783562 | A | G | 0.001 | -0.007 | 9 | 2.166E-09 | 334070 | | 35.821 |
| 115 | rs7862560 | T | C | 0.001 | 0.007 | 9 | 4.048E-08 | 334070 | | 30.127 |
| 116 | rs4877151 | T | C | 0.001 | 0.006 | 9 | 2.998E-08 | 334070 | | 30.710 |
| 117 | rs723968 | C | T | 0.002 | -0.010 | 9 | 9.132E-10 | 334070 | | 37.504 |
| 118 | rs11793831 | T | G | 0.001 | 0.015 | 9 | 1.980E-36 | 334070 | | 158.924 |
| 119 | rs10795831 | T | G | 0.001 | -0.008 | 10 | 4.377E-08 | 334070 | | 29.976 |
| 120 | rs10752262 | T | C | 0.001 | 0.007 | 10 | 1.201E-08 | 334070 | | 32.487 |
| 121 | rs56151069 | T | C | 0.002 | 0.009 | 10 | 1.911E-08 | 334070 | | 31.585 |
| 122 | rs34190234 | C | A | 0.001 | 0.007 | 10 | 9.745E-09 | 334070 | | 32.893 |
| 123 | rs634546 | T | C | 0.001 | -0.007 | 10 | 2.184E-09 | 334070 | | 35.804 |
| 124 | rs7921305 | A | G | 0.001 | 0.010 | 10 | 3.970E-14 | 334070 | | 57.189 |
| 125 | rs2762546 | T | C | 0.001 | -0.008 | 10 | 8.736E-12 | 334070 | | 46.596 |
| 126 | rs35649565 | A | G | 0.001 | 0.008 | 10 | 8.300E-09 | 334070 | | 33.205 |
| 127 | rs12779657 | T | C | 0.001 | 0.008 | 10 | 2.166E-08 | 334070 | | 31.342 |
| 128 | rs12359372 | C | T | 0.001 | 0.007 | 10 | 3.885E-09 | 334070 | | 34.682 |
| 129 | rs2735421 | G | T | 0.001 | -0.010 | 10 | 1.920E-14 | 334070 | | 58.617 |
| 130 | rs55677194 | G | A | 0.002 | 0.013 | 10 | 3.367E-09 | 334070 | | 34.961 |
| 131 | rs12764568 | T | A | 0.001 | -0.006 | 10 | 4.052E-08 | 334070 | | 30.126 |
| 132 | rs2083440 | T | C | 0.001 | 0.007 | 11 | 2.146E-09 | 334070 | | 35.839 |
| 133 | rs478742 | G | A | 0.001 | 0.007 | 11 | 4.222E-09 | 334070 | | 34.521 |
| 134 | rs567203 | C | T | 0.001 | 0.009 | 11 | 3.395E-12 | 334070 | | 48.450 |
| 135 | rs580241 | A | G | 0.001 | 0.009 | 11 | 5.511E-12 | 334070 | | 47.500 |
| 136 | rs12221820 | T | C | 0.001 | -0.007 | 11 | 2.841E-08 | 334070 | | 30.815 |
| 137 | rs4567409 | G | A | 0.001 | -0.006 | 11 | 3.069E-08 | 334070 | | 30.665 |
| 138 | rs7902 | G | A | 0.001 | 0.008 | 11 | 2.794E-12 | 334070 | | 48.832 |
| 139 | rs7965989 | T | G | 0.001 | -0.008 | 12 | 2.598E-10 | 334070 | | 39.958 |
| 140 | rs7966054 | C | T | 0.001 | -0.006 | 12 | 1.833E-08 | 334070 | | 31.666 |
| 141 | rs11171739 | T | C | 0.001 | -0.008 | 12 | 3.340E-11 | 334070 | | 43.969 |
| 142 | rs10129035 | C | T | 0.001 | -0.008 | 12 | 4.876E-11 | 334070 | | 43.229 |
| 143 | rs1727302 | A | G | 0.001 | -0.009 | 12 | 7.454E-12 | 334070 | | 46.908 |
| 144 | rs1333380 | A | G | 0.001 | -0.007 | 13 | 3.740E-08 | 334070 | | 30.281 |
| 145 | rs962490 | A | G | 0.001 | 0.008 | 13 | 3.867E-09 | 334070 | | 34.691 |
| 146 | rs9556958 | T | C | 0.001 | -0.007 | 13 | 5.598E-10 | 334070 | | 38.459 |
| 147 | rs1073242 | A | G | 0.001 | 0.012 | 13 | 1.524E-23 | 334070 | | 100.015 |
| 148 | rs7988627 | A | G | 0.001 | -0.009 | 13 | 1.241E-13 | 334070 | | 54.947 |
| 149 | rs9557427 | T | C | 0.001 | -0.007 | 13 | 2.793E-09 | 334070 | | 35.325 |
| 150 | rs4906348 | T | C | 0.001 | 0.008 | 14 | 2.015E-11 | 334070 | | 44.959 |
| 151 | rs4983187 | T | G | 0.001 | -0.011 | 14 | 5.657E-14 | 334070 | | 56.492 |
| 152 | rs10138733 | G | A | 0.001 | -0.010 | 14 | 6.074E-11 | 334070 | | 42.799 |
| 153 | rs2877774 | C | G | 0.001 | 0.008 | 14 | 3.519E-09 | 334070 | | 34.875 |
| 154 | rs1275202 | C | T | 0.001 | -0.007 | 14 | 1.243E-09 | 334070 | | 36.903 |
| 155 | rs10431632 | A | G | 0.001 | 0.007 | 14 | 1.927E-08 | 334070 | | 31.569 |
| 156 | rs12438542 | G | C | 0.001 | 0.006 | 15 | 2.371E-08 | 334070 | | 31.165 |
| 157 | rs2860049 | T | G | 0.001 | -0.008 | 15 | 2.662E-12 | 334070 | | 48.927 |
| 158 | rs117799466 | C | G | 0.001 | 0.008 | 15 | 4.234E-10 | 334070 | | 39.004 |
| 159 | rs9934839 | G | A | 0.001 | -0.007 | 16 | 6.398E-10 | 334070 | | 38.198 |
| 160 | rs34518736 | G | C | 0.001 | 0.007 | 16 | 5.018E-09 | 334070 | | 34.184 |
| 161 | rs8051058 | C | T | 0.001 | -0.008 | 16 | 2.234E-09 | 334070 | | 35.760 |
| 162 | rs818414 | G | A | 0.001 | 0.008 | 16 | 1.085E-08 | 334070 | | 32.685 |
| 163 | rs61527214 | A | G | 0.001 | 0.007 | 16 | 1.256E-08 | 334070 | | 32.400 |
| 164 | rs62036613 | G | A | 0.001 | -0.008 | 16 | 8.123E-13 | 334070 | | 51.256 |
| 165 | rs2052284 | A | T | 0.001 | 0.007 | 16 | 1.048E-08 | 334070 | | 32.752 |
| 166 | rs72801817 | T | C | 0.001 | 0.008 | 16 | 2.718E-11 | 334070 | | 44.373 |
| 167 | rs9891803 | T | C | 0.001 | -0.007 | 17 | 9.189E-10 | 334070 | | 37.492 |
| 168 | rs11080256 | A | G | 0.001 | 0.007 | 17 | 2.507E-08 | 334070 | | 31.057 |
| 169 | rs62063281 | G | A | 0.001 | -0.010 | 17 | 1.699E-12 | 334070 | | 49.807 |
| 170 | rs10515044 | T | C | 0.001 | 0.006 | 17 | 3.973E-08 | 334070 | | 30.164 |
| 171 | rs1140892 | T | C | 0.003 | 0.020 | 17 | 8.450E-09 | 334070 | | 33.170 |
| 172 | rs303752 | A | G | 0.001 | -0.008 | 18 | 4.573E-11 | 334070 | | 43.355 |
| 173 | rs9960416 | G | A | 0.001 | 0.007 | 18 | 7.037E-09 | 334070 | | 33.526 |
| 174 | rs9964724 | T | C | 0.001 | 0.013 | 18 | 5.972E-25 | 334070 | | 106.435 |
| 175 | rs613872 | T | G | 0.002 | -0.009 | 18 | 4.893E-10 | 334070 | | 38.722 |
| 176 | rs7257285 | G | C | 0.001 | 0.009 | 19 | 7.646E-09 | 334070 | | 33.365 |
| 177 | rs8112975 | G | A | 0.001 | -0.008 | 19 | 3.356E-10 | 334070 | | 39.459 |
| 178 | rs79918827 | A | G | 0.003 | -0.018 | 19 | 1.305E-08 | 334070 | | 32.325 |
| 179 | rs4808787 | G | A | 0.001 | 0.007 | 19 | 2.964E-08 | 334070 | | 30.733 |
| 180 | rs7260359 | T | C | 0.001 | -0.009 | 19 | 1.521E-14 | 334070 | | 59.077 |
| 181 | rs175325 | A | T | 0.001 | -0.007 | 20 | 5.032E-10 | 334070 | | 38.667 |
| 182 | rs1998086 | G | A | 0.001 | -0.009 | 20 | 6.479E-10 | 334070 | | 38.174 |
| 183 | rs67651814 | C | G | 0.001 | -0.009 | 20 | 2.223E-13 | 334070 | | 53.801 |
| 184 | rs4810227 | A | G | 0.001 | 0.009 | 20 | 2.097E-13 | 334070 | | 53.916 |
| 185 | rs28807201 | C | T | 0.001 | 0.007 | 21 | 2.510E-08 | 334070 | | 31.055 |
| 186 | rs11703948 | G | A | 0.002 | 0.013 | 22 | 3.516E-12 | 334070 | | 48.381 |
| 187 | rs9608438 | T | C | 0.001 | 0.008 | 22 | 6.611E-09 | 334070 | | 33.648 |
| 188 | rs5758922 | C | T | 0.001 | -0.006 | 22 | 1.218E-08 | 334070 | | 32.460 |

Supplementary Table 3 IVs of intelligence.

|  | SNP | effect_allele | other_allele | se | beta | chr | pval | samplesize | F |
| --- | --- | --- | --- | --- | --- | --- | --- | --- | --- |
| 1 | rs10917152 | T | C | 0.004 | 0.024 | 1 | 2.227E-09 | 269867 | 35.760 |
| 2 | rs190925241 | A | T | 0.005 | 0.033 | 1 | 9.797E-10 | 269867 | 37.369 |
| 3 | rs10779271 | G | A | 0.003 | -0.016 | 1 | 2.173E-08 | 269867 | 31.338 |
| 4 | rs12035012 | A | C | 0.003 | -0.027 | 1 | 3.675E-16 | 269867 | 66.406 |
| 5 | rs11210871 | G | C | 0.003 | 0.017 | 1 | 3.079E-09 | 269867 | 35.129 |
| 6 | rs12026245 | A | G | 0.003 | -0.018 | 1 | 6.049E-11 | 269867 | 42.811 |
| 7 | rs2678210 | C | T | 0.003 | -0.019 | 1 | 6.970E-10 | 269867 | 38.032 |
| 8 | rs1831539 | C | T | 0.003 | 0.017 | 1 | 4.722E-10 | 269867 | 38.788 |
| 9 | rs2420551 | A | T | 0.004 | -0.029 | 1 | 4.604E-11 | 269867 | 43.336 |
| 10 | rs3128341 | C | T | 0.003 | 0.032 | 1 | 1.632E-20 | 269867 | 86.192 |
| 11 | rs6668048 | T | C | 0.003 | -0.021 | 1 | 4.240E-15 | 269867 | 61.591 |
| 12 | rs600806 | A | G | 0.003 | -0.019 | 1 | 3.571E-10 | 269867 | 39.338 |
| 13 | rs34320898 | C | G | 0.004 | 0.023 | 1 | 2.696E-09 | 269867 | 35.391 |
| 14 | rs112780312 | A | G | 0.003 | -0.018 | 1 | 3.662E-09 | 269867 | 34.798 |
| 15 | rs12470949 | C | T | 0.003 | 0.017 | 2 | 1.323E-08 | 269867 | 32.297 |
| 16 | rs13395129 | C | G | 0.003 | 0.017 | 2 | 1.825E-08 | 269867 | 31.674 |
| 17 | rs62198803 | A | G | 0.003 | 0.019 | 2 | 3.400E-09 | 269867 | 34.940 |
| 18 | rs2955280 | T | C | 0.003 | -0.015 | 2 | 4.900E-08 | 269867 | 29.757 |
| 19 | rs2309812 | T | C | 0.003 | 0.023 | 2 | 9.954E-16 | 269867 | 64.433 |
| 20 | rs4667954 | C | T | 0.003 | -0.017 | 2 | 1.201E-08 | 269867 | 32.490 |
| 21 | rs10189857 | G | A | 0.003 | -0.019 | 2 | 4.907E-12 | 269867 | 47.721 |
| 22 | rs2558096 | G | T | 0.003 | 0.016 | 2 | 1.737E-08 | 269867 | 31.765 |
| 23 | rs297578 | A | G | 0.003 | 0.018 | 2 | 1.728E-09 | 269867 | 36.253 |
| 24 | rs62181012 | C | T | 0.004 | -0.021 | 2 | 1.727E-09 | 269867 | 36.264 |
| 25 | rs7573001 | C | G | 0.003 | -0.016 | 2 | 1.324E-08 | 269867 | 32.297 |
| 26 | rs58593843 | A | G | 0.005 | -0.028 | 2 | 2.666E-09 | 269867 | 35.414 |
| 27 | rs4852252 | C | T | 0.003 | 0.021 | 2 | 3.835E-14 | 269867 | 57.244 |
| 28 | rs11678106 | T | C | 0.003 | 0.016 | 2 | 4.618E-09 | 269867 | 34.339 |
| 29 | rs10189912 | G | A | 0.003 | 0.019 | 2 | 1.219E-11 | 269867 | 45.941 |
| 30 | rs2268894 | T | C | 0.003 | 0.021 | 2 | 3.982E-14 | 269867 | 57.184 |
| 31 | rs13024268 | A | G | 0.003 | -0.017 | 2 | 7.147E-09 | 269867 | 33.490 |
| 32 | rs967569 | T | C | 0.003 | -0.018 | 2 | 8.213E-10 | 269867 | 37.712 |
| 33 | rs11898362 | A | G | 0.003 | -0.018 | 2 | 2.254E-09 | 269867 | 35.737 |
| 34 | rs60262711 | T | C | 0.003 | 0.016 | 2 | 1.652E-08 | 269867 | 31.866 |
| 35 | rs2007176 | C | T | 0.003 | -0.015 | 2 | 2.643E-08 | 269867 | 30.958 |
| 36 | rs35731967 | C | T | 0.004 | -0.022 | 2 | 2.379E-09 | 269867 | 35.641 |
| 37 | rs1589652 | G | A | 0.003 | -0.017 | 3 | 5.823E-10 | 269867 | 38.378 |
| 38 | rs7640196 | T | C | 0.003 | -0.017 | 3 | 3.148E-08 | 269867 | 30.614 |
| 39 | rs11720523 | A | C | 0.003 | 0.018 | 3 | 3.889E-11 | 269867 | 43.666 |
| 40 | rs6770622 | A | G | 0.007 | -0.045 | 3 | 5.761E-11 | 269867 | 42.903 |
| 41 | rs7652296 | G | A | 0.003 | -0.017 | 3 | 3.509E-09 | 269867 | 34.881 |
| 42 | rs13071190 | C | T | 0.003 | -0.018 | 3 | 5.551E-10 | 269867 | 38.477 |
| 43 | rs6550835 | A | G | 0.003 | -0.025 | 3 | 2.439E-17 | 269867 | 71.758 |
| 44 | rs2352974 | T | C | 0.003 | -0.031 | 3 | 3.691E-29 | 269867 | 125.642 |
| 45 | rs3860537 | C | T | 0.003 | -0.019 | 3 | 2.994E-08 | 269867 | 30.714 |
| 46 | rs59142272 | A | G | 0.004 | 0.023 | 3 | 7.319E-10 | 269867 | 37.933 |
| 47 | rs1972860 | A | G | 0.003 | -0.018 | 4 | 2.091E-09 | 269867 | 35.892 |
| 48 | rs2726491 | A | G | 0.003 | -0.028 | 4 | 4.168E-23 | 269867 | 98.010 |
| 49 | rs6535809 | G | A | 0.003 | -0.020 | 4 | 6.650E-13 | 269867 | 51.638 |
| 50 | rs12646225 | T | C | 0.004 | 0.025 | 4 | 2.505E-09 | 269867 | 35.533 |
| 51 | rs17199964 | A | G | 0.006 | -0.039 | 4 | 6.673E-12 | 269867 | 47.114 |
| 52 | rs34811474 | A | G | 0.004 | 0.029 | 4 | 7.148E-16 | 269867 | 65.093 |
| 53 | rs144246 | A | G | 0.003 | 0.015 | 4 | 4.908E-08 | 269867 | 29.757 |
| 54 | rs6819372 | G | A | 0.003 | 0.020 | 4 | 4.017E-13 | 269867 | 52.635 |
| 55 | rs4484297 | C | G | 0.003 | 0.018 | 4 | 7.451E-09 | 269867 | 33.409 |
| 56 | rs67482514 | G | C | 0.003 | 0.018 | 4 | 3.207E-08 | 269867 | 30.581 |
| 57 | rs13165296 | C | A | 0.004 | -0.020 | 5 | 2.435E-08 | 269867 | 31.114 |
| 58 | rs166820 | A | G | 0.004 | 0.024 | 5 | 1.369E-11 | 269867 | 45.711 |
| 59 | rs55763037 | G | A | 0.003 | -0.018 | 5 | 3.743E-08 | 269867 | 30.283 |
| 60 | rs4463213 | A | G | 0.003 | 0.019 | 5 | 3.004E-12 | 269867 | 48.692 |
| 61 | rs34316 | C | A | 0.003 | -0.021 | 5 | 2.818E-14 | 269867 | 57.851 |
| 62 | rs7731260 | A | G | 0.003 | 0.015 | 5 | 2.495E-08 | 269867 | 31.069 |
| 63 | rs6860963 | T | C | 0.003 | 0.020 | 5 | 5.569E-09 | 269867 | 33.977 |
| 64 | rs1840847 | A | G | 0.003 | 0.016 | 5 | 1.440E-08 | 269867 | 32.138 |
| 65 | rs75973558 | G | A | 0.004 | -0.026 | 5 | 9.417E-09 | 269867 | 32.959 |
| 66 | rs36033 | C | T | 0.003 | -0.016 | 5 | 1.017E-08 | 269867 | 32.810 |
| 67 | rs2450333 | A | G | 0.003 | -0.019 | 5 | 1.731E-11 | 269867 | 45.252 |
| 68 | rs1812587 | T | G | 0.003 | -0.017 | 5 | 3.681E-10 | 269867 | 39.275 |
| 69 | rs80170948 | G | T | 0.007 | -0.045 | 5 | 7.686E-10 | 269867 | 37.835 |
| 70 | rs1145123 | C | T | 0.003 | -0.021 | 5 | 1.198E-13 | 269867 | 55.012 |
| 71 | rs405321 | A | G | 0.003 | -0.016 | 5 | 3.318E-08 | 269867 | 30.515 |
| 72 | rs31768 | T | A | 0.003 | -0.018 | 5 | 2.646E-09 | 269867 | 35.426 |
| 73 | rs6903716 | G | A | 0.003 | -0.018 | 6 | 2.387E-09 | 269867 | 35.629 |
| 74 | rs1280049 | C | A | 0.003 | -0.015 | 6 | 3.919E-08 | 269867 | 30.184 |
| 75 | rs12190777 | G | A | 0.003 | -0.017 | 6 | 3.629E-08 | 269867 | 30.338 |
| 76 | rs287879 | G | A | 0.003 | 0.019 | 6 | 8.469E-10 | 269867 | 37.650 |
| 77 | rs566237 | G | A | 0.003 | 0.019 | 6 | 1.817E-10 | 269867 | 40.653 |
| 78 | rs1906252 | A | C | 0.003 | 0.032 | 6 | 7.477E-31 | 269867 | 133.380 |
| 79 | rs9503599 | C | T | 0.003 | 0.017 | 6 | 8.047E-10 | 269867 | 37.749 |
| 80 | rs1233578 | G | A | 0.004 | 0.024 | 6 | 1.075E-09 | 269867 | 37.185 |
| 81 | rs9384679 | T | C | 0.003 | -0.027 | 6 | 7.936E-22 | 269867 | 92.179 |
| 82 | rs13212044 | T | G | 0.003 | -0.018 | 6 | 1.464E-08 | 269867 | 32.103 |
| 83 | rs4725065 | G | A | 0.003 | 0.017 | 7 | 1.518E-09 | 269867 | 36.506 |
| 84 | rs1362739 | A | C | 0.003 | 0.021 | 7 | 1.825E-14 | 269867 | 58.706 |
| 85 | rs799444 | C | T | 0.003 | -0.018 | 7 | 2.481E-11 | 269867 | 44.542 |
| 86 | rs4731392 | G | A | 0.003 | 0.022 | 7 | 2.687E-13 | 269867 | 53.421 |
| 87 | rs115064 | C | T | 0.003 | -0.016 | 7 | 1.065E-08 | 269867 | 32.718 |
| 88 | rs13223152 | G | A | 0.003 | -0.018 | 7 | 2.335E-10 | 269867 | 40.170 |
| 89 | rs56150095 | A | C | 0.003 | -0.022 | 7 | 1.275E-15 | 269867 | 63.952 |
| 90 | rs12535854 | G | C | 0.003 | 0.018 | 7 | 6.732E-10 | 269867 | 38.094 |
| 91 | rs13253386 | G | T | 0.003 | 0.020 | 8 | 2.372E-13 | 269867 | 53.670 |
| 92 | rs13276212 | T | G | 0.003 | 0.015 | 8 | 4.478E-08 | 269867 | 29.932 |
| 93 | rs10954779 | T | C | 0.003 | -0.016 | 8 | 3.038E-09 | 269867 | 35.165 |
| 94 | rs2111490 | G | A | 0.003 | -0.015 | 8 | 1.833E-08 | 269867 | 31.663 |
| 95 | rs4976976 | A | G | 0.003 | 0.017 | 8 | 4.528E-10 | 269867 | 38.875 |
| 96 | rs2920940 | C | T | 0.003 | 0.025 | 8 | 2.759E-14 | 269867 | 57.897 |
| 97 | rs7357604 | G | A | 0.003 | -0.016 | 8 | 2.558E-08 | 269867 | 31.014 |
| 98 | rs2721173 | T | C | 0.003 | -0.016 | 8 | 2.887E-09 | 269867 | 35.260 |
| 99 | rs913264 | T | C | 0.003 | 0.020 | 9 | 7.084E-11 | 269867 | 42.497 |
| 100 | rs11793831 | T | G | 0.003 | 0.028 | 9 | 3.252E-23 | 269867 | 98.506 |
| 101 | rs2987390 | G | C | 0.003 | 0.018 | 9 | 1.192E-08 | 269867 | 32.502 |
| 102 | rs28620532 | G | A | 0.003 | 0.016 | 9 | 1.511E-08 | 269867 | 32.036 |
| 103 | rs702222 | T | C | 0.003 | -0.020 | 9 | 5.019E-12 | 269867 | 47.679 |
| 104 | rs7069887 | C | A | 0.004 | -0.023 | 10 | 7.442E-09 | 269867 | 33.420 |
| 105 | rs2393967 | C | A | 0.003 | 0.019 | 10 | 2.695E-10 | 269867 | 39.879 |
| 106 | rs1408579 | T | C | 0.003 | 0.016 | 10 | 5.230E-09 | 269867 | 34.105 |
| 107 | rs3740422 | C | G | 0.003 | -0.024 | 10 | 1.253E-16 | 269867 | 68.525 |
| 108 | rs35608616 | A | G | 0.003 | -0.018 | 10 | 7.329E-10 | 269867 | 37.933 |
| 109 | rs2508713 | A | T | 0.003 | 0.017 | 11 | 5.916E-09 | 269867 | 33.861 |
| 110 | rs329672 | T | C | 0.003 | 0.017 | 11 | 1.003E-09 | 269867 | 37.320 |
| 111 | rs2885208 | C | T | 0.003 | -0.019 | 11 | 4.579E-08 | 269867 | 29.888 |
| 112 | rs11605348 | A | G | 0.003 | -0.017 | 11 | 9.728E-09 | 269867 | 32.890 |
| 113 | rs7941785 | G | A | 0.003 | -0.016 | 11 | 4.753E-08 | 269867 | 29.811 |
| 114 | rs7116046 | T | C | 0.003 | 0.016 | 11 | 3.267E-08 | 269867 | 30.537 |
| 115 | rs17128425 | A | T | 0.005 | 0.026 | 11 | 1.865E-08 | 269867 | 31.629 |
| 116 | rs2373353 | G | A | 0.003 | 0.016 | 11 | 1.556E-08 | 269867 | 31.979 |
| 117 | rs1962047 | A | G | 0.003 | -0.020 | 12 | 8.892E-12 | 269867 | 46.553 |
| 118 | rs6539284 | C | T | 0.003 | 0.019 | 12 | 5.563E-12 | 269867 | 47.472 |
| 119 | rs1054442 | C | A | 0.003 | 0.021 | 12 | 2.518E-14 | 269867 | 58.080 |
| 120 | rs7312919 | G | C | 0.003 | -0.018 | 12 | 4.828E-10 | 269867 | 38.751 |
| 121 | rs55754731 | C | T | 0.004 | -0.021 | 12 | 6.062E-09 | 269867 | 33.814 |
| 122 | rs1727307 | G | A | 0.003 | -0.018 | 12 | 3.095E-09 | 269867 | 35.117 |
| 123 | rs3843954 | C | G | 0.003 | -0.021 | 13 | 5.305E-10 | 269867 | 38.564 |
| 124 | rs9569206 | G | A | 0.003 | 0.015 | 13 | 4.845E-08 | 269867 | 29.779 |
| 125 | rs2478286 | C | G | 0.003 | -0.026 | 13 | 1.635E-16 | 269867 | 67.997 |
| 126 | rs9516855 | G | A | 0.006 | -0.033 | 13 | 4.193E-08 | 269867 | 30.063 |
| 127 | rs4981713 | G | T | 0.003 | -0.016 | 14 | 7.226E-09 | 269867 | 33.478 |
| 128 | rs12886584 | C | T | 0.004 | -0.021 | 14 | 9.412E-09 | 269867 | 32.959 |
| 129 | rs1007934 | A | G | 0.003 | 0.016 | 14 | 1.004E-08 | 269867 | 32.833 |
| 130 | rs2071407 | C | T | 0.003 | 0.022 | 14 | 1.519E-14 | 269867 | 59.074 |
| 131 | rs2239647 | C | A | 0.003 | 0.021 | 14 | 1.136E-13 | 269867 | 55.116 |
| 132 | rs8006700 | A | T | 0.003 | -0.018 | 14 | 4.964E-10 | 269867 | 38.688 |
| 133 | rs11623436 | T | C | 0.003 | -0.016 | 14 | 9.702E-09 | 269867 | 32.902 |
| 134 | rs17106817 | C | T | 0.003 | -0.017 | 14 | 2.255E-08 | 269867 | 31.259 |
| 135 | rs1369429 | C | T | 0.003 | -0.018 | 15 | 1.146E-09 | 269867 | 37.064 |
| 136 | rs11634187 | G | T | 0.004 | -0.022 | 15 | 1.118E-08 | 269867 | 32.627 |
| 137 | rs7172979 | T | G | 0.009 | 0.061 | 15 | 2.470E-11 | 269867 | 44.556 |
| 138 | rs72739469 | C | T | 0.006 | 0.034 | 15 | 1.181E-09 | 269867 | 37.003 |
| 139 | rs8025964 | A | G | 0.003 | 0.017 | 15 | 5.777E-10 | 269867 | 38.390 |
| 140 | rs8051038 | A | G | 0.003 | 0.019 | 16 | 1.783E-09 | 269867 | 36.192 |
| 141 | rs11076962 | C | T | 0.003 | -0.017 | 16 | 2.574E-08 | 269867 | 31.002 |
| 142 | rs11646221 | T | G | 0.003 | 0.018 | 16 | 1.567E-10 | 269867 | 40.947 |
| 143 | rs2457192 | A | C | 0.003 | -0.020 | 16 | 2.835E-10 | 269867 | 39.791 |
| 144 | rs72768642 | C | T | 0.005 | 0.031 | 16 | 1.456E-08 | 269867 | 32.115 |
| 145 | rs8054299 | G | C | 0.003 | 0.023 | 16 | 3.841E-15 | 269867 | 61.780 |
| 146 | rs2647995 | C | T | 0.003 | 0.020 | 16 | 8.684E-11 | 269867 | 42.094 |
| 147 | rs9888986 | A | G | 0.004 | -0.024 | 16 | 3.517E-08 | 269867 | 30.404 |
| 148 | rs2008514 | A | G | 0.003 | -0.029 | 16 | 1.246E-24 | 269867 | 104.960 |
| 149 | rs2285640 | A | G | 0.003 | 0.018 | 17 | 2.376E-10 | 269867 | 40.132 |
| 150 | rs4793161 | G | A | 0.003 | 0.018 | 17 | 4.969E-08 | 269867 | 29.724 |
| 151 | rs11079849 | T | C | 0.003 | 0.017 | 17 | 2.260E-08 | 269867 | 31.259 |
| 152 | rs66954617 | G | A | 0.003 | 0.021 | 17 | 1.722E-13 | 269867 | 54.302 |
| 153 | rs17698176 | G | T | 0.004 | 0.020 | 17 | 1.702E-08 | 269867 | 31.809 |
| 154 | rs6508220 | G | A | 0.003 | 0.023 | 18 | 9.561E-17 | 269867 | 69.056 |
| 155 | rs889169 | A | G | 0.003 | 0.016 | 19 | 2.751E-08 | 269867 | 30.880 |
| 156 | rs2072490 | T | C | 0.003 | 0.017 | 19 | 5.930E-10 | 269867 | 38.341 |
| 157 | rs7248006 | C | T | 0.003 | 0.019 | 19 | 1.054E-11 | 269867 | 46.226 |
| 158 | rs73068339 | C | G | 0.003 | 0.019 | 19 | 5.955E-10 | 269867 | 38.341 |
| 159 | rs17002025 | A | G | 0.004 | 0.026 | 19 | 1.888E-09 | 269867 | 36.084 |
| 160 | rs144026674 | T | C | 0.007 | 0.041 | 19 | 3.190E-08 | 269867 | 30.592 |
| 161 | rs78084033 | C | A | 0.004 | 0.023 | 20 | 1.617E-08 | 269867 | 31.911 |
| 162 | rs6019535 | A | G | 0.003 | 0.025 | 20 | 3.284E-17 | 269867 | 71.166 |
| 163 | rs2836921 | A | G | 0.003 | 0.020 | 21 | 6.537E-12 | 269867 | 47.156 |
| 164 | rs4821995 | G | A | 0.003 | -0.016 | 22 | 2.616E-08 | 269867 | 30.969 |
| 165 | rs5750830 | A | C | 0.003 | 0.023 | 22 | 2.459E-13 | 269867 | 53.597 |

Supplementary Table 4 IVs of income.

|  | SNP | effect_allele | other_allele | se | beta | chr | pval | samplesize | F |
| --- | --- | --- | --- | --- | --- | --- | --- | --- | --- |
| 1 | rs488786 | T | C | 0.004 | 0.021 | 1 | 6.700E-09 | 397751 | 33.626 |
| 2 | rs6699397 | G | A | 0.003 | -0.019 | 1 | 3.500E-12 | 397751 | 48.414 |
| 3 | rs11165472 | T | A | 0.003 | -0.015 | 1 | 8.000E-09 | 397751 | 33.282 |
| 4 | rs2820314 | C | A | 0.003 | -0.017 | 1 | 2.900E-09 | 397751 | 35.267 |
| 5 | rs11588857 | A | G | 0.003 | 0.021 | 1 | 4.900E-11 | 397751 | 43.218 |
| 6 | rs6429636 | T | G | 0.003 | 0.019 | 1 | 5.000E-11 | 397751 | 43.172 |
| 7 | rs12692596 | T | C | 0.003 | -0.015 | 2 | 1.700E-08 | 397751 | 31.779 |
| 8 | rs11678501 | C | T | 0.009 | -0.052 | 2 | 1.200E-09 | 397751 | 36.984 |
| 9 | rs1455350 | A | T | 0.003 | -0.017 | 2 | 9.200E-11 | 397751 | 41.990 |
| 10 | rs62183028 | T | G | 0.003 | -0.019 | 2 | 3.200E-11 | 397751 | 44.021 |
| 11 | rs387780 | C | T | 0.003 | 0.017 | 2 | 3.300E-09 | 397751 | 35.014 |
| 12 | rs13002946 | A | T | 0.003 | 0.021 | 2 | 7.300E-12 | 397751 | 46.939 |
| 13 | rs2332719 | G | A | 0.003 | -0.018 | 3 | 5.700E-10 | 397751 | 38.416 |
| 14 | rs11714337 | A | G | 0.003 | 0.015 | 3 | 9.400E-09 | 397751 | 32.966 |
| 15 | rs11917431 | T | C | 0.003 | 0.023 | 3 | 3.100E-15 | 397751 | 62.190 |
| 16 | rs1229984 | C | T | 0.008 | -0.049 | 4 | 1.000E-09 | 397751 | 37.233 |
| 17 | rs7700107 | C | A | 0.004 | -0.023 | 4 | 2.500E-09 | 397751 | 35.532 |
| 18 | rs32940 | C | T | 0.003 | 0.021 | 5 | 2.900E-13 | 397751 | 53.285 |
| 19 | rs6868457 | C | T | 0.003 | 0.021 | 5 | 3.100E-15 | 397751 | 62.202 |
| 20 | rs2515919 | G | A | 0.003 | -0.016 | 6 | 5.700E-09 | 397751 | 33.951 |
| 21 | rs9388490 | T | C | 0.003 | 0.015 | 6 | 1.500E-08 | 397751 | 32.038 |
| 22 | rs3130264 | G | C | 0.003 | -0.015 | 6 | 5.100E-09 | 397751 | 34.159 |
| 23 | rs73015322 | T | G | 0.005 | -0.027 | 6 | 4.100E-08 | 397751 | 30.117 |
| 24 | rs968050 | T | C | 0.003 | 0.022 | 6 | 3.100E-17 | 397751 | 71.302 |
| 25 | rs71576284 | A | C | 0.015 | -0.084 | 7 | 3.300E-08 | 397751 | 30.550 |
| 26 | rs77126132 | A | G | 0.005 | 0.027 | 7 | 5.200E-09 | 397751 | 34.120 |
| 27 | rs12531825 | A | G | 0.004 | -0.026 | 7 | 2.100E-10 | 397751 | 40.403 |
| 28 | rs1421334 | C | A | 0.003 | 0.016 | 8 | 1.000E-09 | 397751 | 37.238 |
| 29 | rs2068428 | T | C | 0.003 | 0.017 | 9 | 4.200E-08 | 397751 | 30.069 |
| 30 | rs10429582 | C | T | 0.003 | 0.027 | 9 | 7.600E-24 | 397751 | 101.388 |
| 31 | rs10761035 | A | G | 0.003 | 0.019 | 9 | 3.700E-08 | 397751 | 30.322 |
| 32 | rs7896518 | G | A | 0.003 | 0.015 | 10 | 4.100E-08 | 397751 | 30.089 |
| 33 | rs11191116 | T | C | 0.003 | -0.016 | 10 | 3.500E-09 | 397751 | 34.888 |
| 34 | rs34473884 | A | G | 0.003 | 0.017 | 10 | 1.700E-08 | 397751 | 31.769 |
| 35 | rs589914 | G | A | 0.003 | 0.016 | 11 | 4.400E-08 | 397751 | 29.983 |
| 36 | rs9556958 | T | C | 0.003 | -0.015 | 13 | 8.500E-09 | 397751 | 33.159 |
| 37 | rs1239705 | G | A | 0.003 | 0.016 | 13 | 4.700E-09 | 397751 | 34.291 |
| 38 | rs12883788 | T | C | 0.003 | -0.019 | 14 | 1.400E-12 | 397751 | 50.141 |
| 39 | rs4115668 | A | G | 0.003 | -0.018 | 16 | 1.100E-10 | 397751 | 41.557 |
| 40 | rs9891103 | T | C | 0.003 | -0.023 | 17 | 9.399E-14 | 397751 | 55.492 |
| 41 | rs11665242 | G | A | 0.003 | -0.018 | 18 | 6.001E-11 | 397751 | 42.819 |
| 42 | rs11877758 | G | T | 0.003 | -0.020 | 18 | 1.200E-12 | 397751 | 50.433 |
| 43 | rs784256 | A | G | 0.003 | -0.025 | 18 | 7.900E-14 | 397751 | 55.831 |
| 44 | rs75413320 | C | T | 0.004 | -0.026 | 19 | 9.500E-10 | 397751 | 37.428 |
| 45 | rs2362523 | G | A | 0.003 | 0.016 | 19 | 1.400E-08 | 397751 | 32.149 |
| 46 | rs2422859 | G | T | 0.003 | 0.016 | 20 | 6.200E-10 | 397751 | 38.246 |
| 47 | rs6035877 | C | A | 0.003 | -0.015 | 20 | 4.300E-08 | 397751 | 29.987 |
| 48 | rs5754738 | G | A | 0.003 | -0.016 | 22 | 2.700E-08 | 397751 | 30.948 |

Supplementary Table 5 IVs of social deprivation.

|  | SNP | effect_allele | other_allele | se | beta | chr | pval | samplesize | | F |
| --- | --- | --- | --- | --- | --- | --- | --- | --- | --- | --- |
| 1 | rs56142341 | C | G | 0.004 | -0.026 | 1 | 5.200E-09 | 462464 | 34.121 | |
| 2 | rs1947083 | A | G | 0.002 | 0.012 | 1 | 1.900E-08 | 462464 | 31.612 | |
| 3 | rs12133063 | A | C | 0.002 | 0.015 | 1 | 6.100E-12 | 462464 | 47.302 | |
| 4 | rs989532 | G | A | 0.002 | 0.014 | 2 | 7.399E-11 | 462464 | 42.414 | |
| 5 | rs1483246 | C | T | 0.002 | -0.012 | 2 | 3.100E-09 | 462464 | 35.100 | |
| 6 | rs2403326 | G | A | 0.002 | 0.012 | 5 | 1.200E-08 | 462464 | 32.447 | |
| 7 | rs253125 | C | T | 0.002 | 0.013 | 5 | 1.900E-08 | 462464 | 31.609 | |
| 8 | rs78257128 | T | C | 0.003 | -0.017 | 5 | 2.400E-09 | 462464 | 35.578 | |
| 9 | rs12203592 | T | C | 0.002 | 0.017 | 6 | 1.700E-12 | 462464 | 49.767 | |
| 10 | rs7740440 | A | G | 0.002 | 0.012 | 6 | 5.700E-09 | 462464 | 33.928 | |
| 11 | rs6931604 | T | C | 0.002 | -0.012 | 6 | 4.100E-09 | 462464 | 34.577 | |
| 12 | rs62477310 | C | T | 0.002 | -0.012 | 7 | 5.400E-09 | 462464 | 34.022 | |
| 13 | rs990706 | T | C | 0.003 | 0.016 | 11 | 7.900E-09 | 462464 | 33.292 | |
| 14 | rs704067 | A | G | 0.002 | 0.012 | 12 | 3.900E-09 | 462464 | 34.675 | |
| 15 | rs113345285 | C | T | 0.003 | 0.016 | 14 | 3.200E-08 | 462464 | 30.558 | |
| 16 | rs3865018 | T | C | 0.002 | -0.013 | 15 | 4.200E-09 | 462464 | 34.519 | |
| 17 | rs11855821 | A | G | 0.002 | -0.013 | 15 | 3.900E-08 | 462464 | 30.197 | |
| 18 | rs4785187 | A | G | 0.002 | 0.014 | 16 | 1.500E-08 | 462464 | 32.064 | |

Supplementary Table 6 IVs of attention-deficit hyperactivity disorder.

|  | SNP | effect_allele | other_allele | se | beta | chr | pval | samplesize | F |
| --- | --- | --- | --- | --- | --- | --- | --- | --- | --- |
| 1 | rs112984125 | A | G | 0.015 | -0.106 | 1 | 3.581E-13 | 55374 | 52.717 |
| 2 | rs1222063 | A | G | 0.017 | 0.096 | 1 | 3.068E-08 | 55374 | 30.567 |
| 3 | rs9677504 | A | G | 0.021 | 0.117 | 2 | 1.391E-08 | 55374 | 32.205 |
| 4 | rs4858241 | G | T | 0.014 | -0.079 | 3 | 1.740E-08 | 55374 | 31.764 |
| 5 | rs28411770 | C | T | 0.015 | -0.086 | 4 | 1.152E-08 | 55374 | 32.516 |
| 6 | rs4916723 | C | A | 0.014 | 0.077 | 5 | 1.576E-08 | 55374 | 32.195 |
| 7 | rs10262192 | A | G | 0.013 | 0.073 | 7 | 2.887E-08 | 55374 | 30.755 |
| 8 | rs74760947 | G | A | 0.032 | 0.180 | 8 | 1.348E-08 | 55374 | 32.170 |
| 9 | rs11591402 | A | T | 0.016 | -0.093 | 10 | 1.336E-08 | 55374 | 32.092 |
| 10 | rs1427829 | G | A | 0.013 | -0.080 | 12 | 1.822E-09 | 55374 | 36.091 |
| 11 | rs281324 | C | T | 0.013 | 0.074 | 15 | 2.678E-08 | 55374 | 30.908 |
| 12 | rs212178 | A | G | 0.020 | -0.115 | 16 | 7.677E-09 | 55374 | 33.293 |

Supplementary Table 7 IVs of bipolar disorder.

|  | SNP | effect_allele | other_allele | se | beta | chr | pval | samplesize | F |
| --- | --- | --- | --- | --- | --- | --- | --- | --- | --- |
| 1 | rs2314398 | G | C | 0.014 | -0.084 | 2 | 5.920E-09 | 51710 | 34.110 |
| 2 | rs9834970 | C | T | 0.013 | 0.101 | 3 | 5.531E-14 | 51710 | 56.814 |
| 3 | rs2071044 | T | C | 0.014 | -0.078 | 3 | 9.092E-09 | 51710 | 33.128 |
| 4 | rs11724116 | T | C | 0.019 | -0.104 | 4 | 3.267E-08 | 51710 | 30.658 |
| 5 | rs329319 | G | A | 0.014 | -0.079 | 5 | 1.539E-08 | 51710 | 32.140 |
| 6 | rs55648125 | G | A | 0.022 | 0.117 | 6 | 4.922E-08 | 51710 | 29.662 |
| 7 | rs10455979 | G | C | 0.014 | 0.075 | 6 | 4.596E-08 | 51710 | 29.973 |
| 8 | rs17150022 | C | T | 0.020 | 0.113 | 7 | 2.701E-08 | 51710 | 30.793 |
| 9 | rs13231398 | C | G | 0.022 | -0.121 | 7 | 3.361E-08 | 51710 | 30.376 |
| 10 | rs73496688 | A | T | 0.019 | 0.109 | 11 | 1.047E-08 | 51710 | 32.732 |
| 11 | rs174592 | G | A | 0.014 | 0.077 | 11 | 3.664E-08 | 51710 | 30.133 |
| 12 | rs10744560 | T | C | 0.014 | 0.083 | 12 | 2.918E-09 | 51710 | 35.318 |
| 13 | rs71395455 | G | A | 0.015 | -0.082 | 15 | 1.934E-08 | 51710 | 31.618 |
| 14 | rs884301 | T | C | 0.014 | 0.080 | 17 | 5.801E-09 | 51710 | 33.857 |
| 15 | rs111444407 | T | C | 0.018 | 0.117 | 19 | 2.404E-10 | 51710 | 40.157 |
| 16 | rs5758065 | G | C | 0.014 | -0.074 | 22 | 3.228E-08 | 51710 | 30.374 |

Supplementary Table 8 IVs of major depressive disorder.

|  | SNP | effect_allele | other_allele | se | beta | chr | pval | samplesize | F |
| --- | --- | --- | --- | --- | --- | --- | --- | --- | --- |
| 1 | rs159963 | A | C | 0.005 | -0.027 | 1 | 3.193E-08 | 480359 | 30.365 |
| 2 | rs1432639 | A | C | 0.005 | 0.039 | 1 | 4.553E-15 | 480359 | 60.838 |
| 3 | rs2389016 | T | C | 0.005 | 0.031 | 1 | 1.018E-08 | 480359 | 33.117 |
| 4 | rs9427672 | G | A | 0.006 | 0.032 | 1 | 3.119E-08 | 480359 | 30.630 |
| 5 | rs1226412 | T | C | 0.006 | 0.033 | 2 | 2.384E-08 | 480359 | 31.669 |
| 6 | rs76485002 | G | A | 0.018 | -0.109 | 2 | 1.603E-09 | 480359 | 36.470 |
| 7 | rs11682175 | C | T | 0.005 | 0.028 | 2 | 4.680E-09 | 480359 | 34.274 |
| 8 | rs7430565 | A | G | 0.005 | -0.029 | 3 | 2.868E-09 | 480359 | 36.002 |
| 9 | rs34215985 | G | C | 0.006 | 0.037 | 4 | 3.132E-09 | 480359 | 35.048 |
| 10 | rs11135349 | C | A | 0.005 | 0.029 | 5 | 1.092E-09 | 480359 | 37.510 |
| 11 | rs247910 | G | A | 0.005 | 0.032 | 5 | 1.065E-10 | 480359 | 41.329 |
| 12 | rs1363104 | G | C | 0.005 | -0.031 | 5 | 7.376E-11 | 480359 | 42.798 |
| 13 | rs9402472 | A | G | 0.006 | 0.033 | 6 | 2.781E-08 | 480359 | 30.717 |
| 14 | rs6905391 | A | G | 0.007 | -0.044 | 6 | 1.348E-10 | 480359 | 41.214 |
| 15 | rs10950398 | A | G | 0.005 | 0.027 | 7 | 2.548E-08 | 480359 | 31.494 |
| 16 | rs12666117 | A | G | 0.005 | 0.027 | 7 | 1.346E-08 | 480359 | 32.588 |
| 17 | rs1354115 | A | C | 0.005 | 0.028 | 9 | 2.370E-08 | 480359 | 31.717 |
| 18 | rs10959913 | G | T | 0.006 | -0.033 | 9 | 5.062E-09 | 480359 | 34.327 |
| 19 | rs7856424 | T | C | 0.005 | -0.031 | 9 | 8.479E-09 | 480359 | 33.342 |
| 20 | rs61867293 | T | C | 0.006 | -0.037 | 10 | 6.966E-10 | 480359 | 37.593 |
| 21 | rs1806153 | T | G | 0.006 | 0.036 | 11 | 1.178E-09 | 480359 | 37.439 |
| 22 | rs4074723 | C | A | 0.005 | 0.027 | 12 | 3.118E-08 | 480359 | 30.365 |
| 23 | rs12552 | G | A | 0.005 | -0.043 | 13 | 6.072E-19 | 480359 | 79.866 |
| 24 | rs10149470 | G | A | 0.005 | 0.029 | 14 | 3.054E-09 | 480359 | 35.018 |
| 25 | rs4904738 | C | T | 0.005 | 0.029 | 14 | 2.572E-09 | 480359 | 34.795 |
| 26 | rs915057 | G | A | 0.005 | 0.030 | 14 | 7.609E-10 | 480359 | 37.473 |
| 27 | rs2005864 | T | C | 0.005 | 0.028 | 14 | 6.731E-09 | 480359 | 33.118 |
| 28 | rs8025231 | C | A | 0.005 | 0.034 | 15 | 2.358E-12 | 480359 | 49.873 |
| 29 | rs7198928 | C | T | 0.005 | -0.028 | 16 | 1.004E-08 | 480359 | 32.269 |
| 30 | rs11643192 | A | C | 0.005 | 0.027 | 16 | 3.359E-08 | 480359 | 30.367 |
| 31 | rs8063603 | A | G | 0.005 | -0.031 | 16 | 6.865E-09 | 480359 | 33.770 |
| 32 | rs17727765 | C | T | 0.009 | 0.051 | 17 | 8.513E-09 | 480359 | 33.323 |
| 33 | rs12958048 | G | A | 0.005 | -0.034 | 18 | 3.613E-11 | 480359 | 43.929 |
| 34 | rs11663393 | A | G | 0.005 | 0.028 | 18 | 1.645E-08 | 480359 | 32.188 |
| 35 | rs62099069 | T | A | 0.005 | 0.028 | 18 | 1.311E-08 | 480359 | 32.410 |
| 36 | rs5758265 | A | G | 0.005 | 0.031 | 22 | 7.554E-09 | 480359 | 32.965 |

Supplementary Table 9 IVs of schizophrenia.

|  | SNP | effect_allele | other_allele | se | beta | chr | pval | samplesize | F |
| --- | --- | --- | --- | --- | --- | --- | --- | --- | --- |
| 1 | rs3900555 | A | G | 0.008 | 0.043 | 1 | 9.947E-09 | 175799 | 32.714 |
| 2 | rs10779702 | G | A | 0.008 | -0.053 | 1 | 3.219E-10 | 175799 | 39.356 |
| 3 | rs56335113 | G | A | 0.008 | -0.065 | 1 | 1.061E-15 | 175799 | 64.993 |
| 4 | rs6673880 | G | A | 0.008 | 0.056 | 1 | 1.556E-11 | 175799 | 45.520 |
| 5 | rs10157075 | T | A | 0.010 | -0.059 | 1 | 5.264E-09 | 175799 | 34.129 |
| 6 | rs12039854 | G | T | 0.009 | 0.055 | 1 | 2.571E-09 | 175799 | 35.870 |
| 7 | rs7515363 | T | C | 0.008 | -0.049 | 1 | 3.425E-09 | 175799 | 35.001 |
| 8 | rs61787564 | A | G | 0.011 | -0.064 | 1 | 1.339E-08 | 175799 | 32.481 |
| 9 | rs2970610 | C | T | 0.008 | -0.064 | 1 | 2.507E-16 | 175799 | 67.122 |
| 10 | rs12042444 | T | C | 0.008 | -0.064 | 1 | 8.533E-17 | 175799 | 69.729 |
| 11 | rs12126806 | T | C | 0.009 | -0.050 | 1 | 6.912E-09 | 175799 | 33.531 |
| 12 | rs11587347 | G | C | 0.014 | 0.102 | 1 | 4.000E-14 | 175799 | 57.422 |
| 13 | rs558120 | C | T | 0.011 | 0.058 | 1 | 3.323E-08 | 175799 | 30.456 |
| 14 | rs6588168 | T | C | 0.008 | 0.046 | 1 | 1.167E-09 | 175799 | 37.450 |
| 15 | rs11165846 | G | C | 0.008 | 0.054 | 1 | 6.452E-11 | 175799 | 42.963 |
| 16 | rs4950119 | C | A | 0.010 | 0.091 | 1 | 8.364E-19 | 175799 | 78.902 |
| 17 | rs12138231 | A | T | 0.011 | 0.066 | 1 | 7.372E-10 | 175799 | 37.686 |
| 18 | rs10559 | G | A | 0.008 | 0.044 | 1 | 4.024E-08 | 175799 | 30.385 |
| 19 | rs60124939 | T | C | 0.009 | -0.053 | 1 | 3.764E-09 | 175799 | 34.543 |
| 20 | rs16851048 | C | T | 0.009 | 0.068 | 1 | 8.279E-14 | 175799 | 55.998 |
| 21 | rs1486472 | A | G | 0.008 | 0.057 | 1 | 9.156E-13 | 175799 | 51.335 |
| 22 | rs3770752 | G | A | 0.008 | -0.059 | 2 | 1.741E-12 | 175799 | 49.831 |
| 23 | rs2139054 | C | A | 0.008 | 0.072 | 2 | 1.045E-20 | 175799 | 86.474 |
| 24 | rs62152282 | T | A | 0.009 | 0.058 | 2 | 6.396E-10 | 161405 | 38.197 |
| 25 | rs2167378 | T | C | 0.008 | -0.058 | 2 | 9.901E-15 | 175799 | 59.193 |
| 26 | rs6715416 | G | A | 0.008 | -0.060 | 2 | 1.049E-15 | 175799 | 64.007 |
| 27 | rs34181670 | C | T | 0.009 | -0.049 | 2 | 1.128E-08 | 175799 | 32.691 |
| 28 | rs999494 | T | C | 0.010 | -0.057 | 2 | 3.618E-09 | 175799 | 34.886 |
| 29 | rs62183855 | C | A | 0.010 | -0.061 | 2 | 1.970E-09 | 175799 | 35.766 |
| 30 | rs10173857 | T | C | 0.009 | -0.050 | 2 | 3.836E-08 | 175799 | 30.315 |
| 31 | rs778371 | G | A | 0.009 | 0.070 | 2 | 2.800E-16 | 175799 | 66.855 |
| 32 | rs17247190 | G | A | 0.009 | 0.052 | 2 | 2.152E-08 | 175799 | 31.584 |
| 33 | rs1396728 | T | C | 0.008 | -0.048 | 2 | 1.628E-09 | 175799 | 36.609 |
| 34 | rs7582445 | C | A | 0.008 | -0.045 | 2 | 8.969E-09 | 175799 | 32.588 |
| 35 | rs6546857 | G | A | 0.009 | 0.054 | 2 | 8.702E-09 | 175799 | 33.250 |
| 36 | rs6430491 | A | G | 0.009 | -0.054 | 2 | 6.705E-10 | 175799 | 38.519 |
| 37 | rs12991836 | C | A | 0.008 | 0.056 | 2 | 8.190E-13 | 175799 | 50.633 |
| 38 | rs60617652 | A | G | 0.008 | -0.046 | 2 | 9.996E-10 | 175799 | 36.948 |
| 39 | rs11680723 | G | C | 0.010 | 0.084 | 2 | 1.052E-16 | 175799 | 68.297 |
| 40 | rs7604885 | A | G | 0.008 | 0.046 | 2 | 5.942E-09 | 175799 | 33.488 |
| 41 | rs11693094 | T | C | 0.008 | -0.056 | 2 | 3.201E-13 | 175799 | 53.330 |
| 42 | rs2033996 | A | T | 0.010 | 0.052 | 2 | 4.326E-08 | 175799 | 29.960 |
| 43 | rs68002929 | A | T | 0.010 | 0.072 | 2 | 5.518E-13 | 175799 | 51.520 |
| 44 | rs1881046 | T | G | 0.008 | -0.044 | 2 | 1.920E-08 | 175799 | 31.587 |
| 45 | rs13090130 | A | G | 0.008 | -0.052 | 3 | 1.037E-11 | 175799 | 45.780 |
| 46 | rs11129383 | A | G | 0.008 | 0.045 | 3 | 3.977E-08 | 175799 | 30.379 |
| 47 | rs4678552 | A | G | 0.009 | 0.054 | 3 | 3.192E-10 | 175799 | 39.138 |
| 48 | rs167924 | G | A | 0.009 | 0.054 | 3 | 1.844E-10 | 175799 | 40.363 |
| 49 | rs10935184 | C | T | 0.008 | -0.061 | 3 | 7.934E-15 | 175799 | 60.955 |
| 50 | rs17194490 | T | G | 0.011 | 0.076 | 3 | 1.532E-11 | 175799 | 45.706 |
| 51 | rs1399551 | A | T | 0.008 | 0.054 | 3 | 4.506E-11 | 175799 | 43.847 |
| 52 | rs1604060 | G | A | 0.013 | 0.076 | 3 | 1.553E-08 | 175799 | 32.000 |
| 53 | rs525297 | C | A | 0.008 | -0.046 | 3 | 5.806E-09 | 161405 | 33.899 |
| 54 | rs7647398 | T | C | 0.010 | -0.083 | 3 | 2.455E-17 | 175799 | 71.553 |
| 55 | rs1866862 | T | A | 0.008 | 0.050 | 3 | 8.498E-11 | 175799 | 42.005 |
| 56 | rs6804677 | C | T | 0.008 | 0.051 | 3 | 3.838E-10 | 175799 | 39.022 |
| 57 | rs2710323 | C | T | 0.007 | -0.072 | 3 | 3.175E-22 | 175799 | 93.873 |
| 58 | rs59971314 | C | G | 0.008 | 0.046 | 3 | 5.877E-09 | 175799 | 34.047 |
| 59 | rs59518103 | C | T | 0.014 | -0.078 | 4 | 4.513E-08 | 175799 | 29.828 |
| 60 | rs215483 | A | G | 0.008 | 0.047 | 4 | 4.228E-09 | 175799 | 34.666 |
| 61 | rs61405217 | T | C | 0.008 | -0.051 | 4 | 1.433E-11 | 175799 | 45.026 |
| 62 | rs35734242 | C | T | 0.008 | 0.051 | 4 | 2.238E-11 | 175799 | 44.558 |
| 63 | rs13107325 | T | C | 0.017 | 0.157 | 4 | 1.919E-21 | 175799 | 90.194 |
| 64 | rs28521069 | T | C | 0.008 | 0.046 | 4 | 4.685E-09 | 175799 | 34.497 |
| 65 | rs1454606 | T | C | 0.010 | 0.063 | 4 | 5.002E-10 | 175799 | 38.386 |
| 66 | rs28454198 | C | G | 0.008 | -0.048 | 4 | 5.972E-10 | 175799 | 38.535 |
| 67 | rs28482891 | A | C | 0.008 | 0.043 | 4 | 4.006E-08 | 175799 | 29.758 |
| 68 | rs7681616 | A | C | 0.009 | -0.049 | 4 | 2.703E-08 | 175799 | 30.884 |
| 69 | rs12498839 | A | G | 0.015 | 0.117 | 4 | 3.239E-15 | 175799 | 61.761 |
| 70 | rs13159353 | C | G | 0.008 | -0.043 | 5 | 2.772E-08 | 175799 | 30.815 |
| 71 | rs10117 | A | G | 0.008 | -0.053 | 5 | 1.885E-11 | 175799 | 44.832 |
| 72 | rs10035564 | G | A | 0.008 | 0.059 | 5 | 1.704E-14 | 175799 | 58.180 |
| 73 | rs7701188 | A | G | 0.012 | -0.075 | 5 | 2.814E-10 | 175799 | 40.074 |
| 74 | rs252812 | G | A | 0.009 | -0.053 | 5 | 5.772E-09 | 175799 | 33.788 |
| 75 | rs3733710 | T | C | 0.008 | -0.045 | 5 | 5.517E-09 | 161405 | 33.998 |
| 76 | rs7709645 | C | G | 0.008 | 0.065 | 5 | 6.109E-17 | 175799 | 69.439 |
| 77 | rs11241041 | T | G | 0.010 | 0.060 | 5 | 5.350E-10 | 161405 | 38.545 |
| 78 | rs72723227 | A | G | 0.008 | -0.050 | 5 | 3.764E-10 | 175799 | 39.538 |
| 79 | rs11740474 | T | A | 0.008 | 0.048 | 5 | 4.426E-09 | 175799 | 34.843 |
| 80 | rs12652777 | C | T | 0.008 | -0.044 | 5 | 5.102E-09 | 175799 | 34.130 |
| 81 | rs9687282 | G | T | 0.008 | 0.048 | 5 | 7.338E-09 | 175799 | 33.689 |
| 82 | rs72802887 | G | A | 0.009 | -0.067 | 5 | 8.529E-13 | 175799 | 51.287 |
| 83 | rs9470670 | G | T | 0.010 | 0.056 | 6 | 1.908E-08 | 175799 | 31.538 |
| 84 | rs2206956 | A | G | 0.008 | 0.045 | 6 | 4.040E-09 | 175799 | 34.600 |
| 85 | rs34555420 | T | G | 0.017 | -0.169 | 6 | 1.823E-22 | 161405 | 95.086 |
| 86 | rs6938026 | G | A | 0.009 | -0.060 | 6 | 7.233E-12 | 175799 | 46.961 |
| 87 | rs6925079 | C | T | 0.008 | 0.047 | 6 | 1.812E-09 | 175799 | 36.477 |
| 88 | rs80249955 | T | C | 0.028 | 0.155 | 6 | 4.629E-08 | 165729 | 29.842 |
| 89 | rs9487653 | G | A | 0.010 | -0.055 | 6 | 4.399E-08 | 175799 | 30.083 |
| 90 | rs140365013 | A | G | 0.016 | -0.206 | 6 | 1.205E-39 | 175799 | 173.861 |
| 91 | rs9459170 | C | T | 0.009 | -0.051 | 6 | 3.832E-08 | 175799 | 30.193 |
| 92 | rs55648125 | G | A | 0.013 | 0.072 | 6 | 4.424E-08 | 175799 | 29.753 |
| 93 | rs217310 | T | A | 0.008 | -0.049 | 6 | 9.936E-11 | 175799 | 42.171 |
| 94 | rs12190758 | G | A | 0.009 | 0.061 | 6 | 6.015E-11 | 175799 | 42.529 |
| 95 | rs9398171 | T | C | 0.008 | -0.050 | 6 | 4.481E-10 | 175799 | 38.568 |
| 96 | rs58120505 | C | T | 0.008 | -0.083 | 7 | 6.265E-28 | 175799 | 121.004 |
| 97 | rs6946576 | A | G | 0.008 | -0.046 | 7 | 4.936E-08 | 175799 | 29.598 |
| 98 | rs13233308 | T | C | 0.008 | -0.046 | 7 | 1.230E-09 | 175799 | 37.299 |
| 99 | rs7803571 | T | C | 0.008 | -0.058 | 7 | 4.488E-14 | 175799 | 57.438 |
| 100 | rs12671608 | C | T | 0.011 | 0.060 | 7 | 4.440E-08 | 175799 | 30.198 |
| 101 | rs2944821 | C | G | 0.008 | -0.049 | 7 | 2.509E-10 | 175799 | 40.110 |
| 102 | rs1593304 | G | A | 0.010 | 0.063 | 7 | 5.914E-11 | 175799 | 42.445 |
| 103 | rs7779548 | A | G | 0.008 | -0.071 | 7 | 1.821E-19 | 175799 | 81.449 |
| 104 | rs35274762 | C | T | 0.012 | -0.102 | 7 | 5.910E-17 | 175799 | 70.318 |
| 105 | rs56226048 | T | G | 0.011 | 0.061 | 7 | 3.086E-08 | 175799 | 30.909 |
| 106 | rs2349487 | T | C | 0.008 | 0.046 | 7 | 5.710E-09 | 175799 | 34.184 |
| 107 | rs79210963 | C | T | 0.013 | 0.084 | 7 | 1.859E-11 | 175799 | 44.757 |
| 108 | rs2470951 | T | A | 0.008 | 0.053 | 7 | 2.715E-12 | 175799 | 49.561 |
| 109 | rs211824 | A | G | 0.008 | -0.048 | 7 | 4.513E-10 | 175799 | 39.029 |
| 110 | rs73229090 | A | C | 0.014 | -0.098 | 8 | 2.443E-12 | 175799 | 48.798 |
| 111 | rs74804370 | C | T | 0.022 | 0.120 | 8 | 3.684E-08 | 165729 | 30.199 |
| 112 | rs1434281 | A | G | 0.008 | -0.045 | 8 | 1.083E-08 | 175799 | 32.346 |
| 113 | rs4129585 | C | A | 0.008 | -0.065 | 8 | 2.506E-17 | 175799 | 71.488 |
| 114 | rs59498392 | G | C | 0.009 | -0.057 | 8 | 1.104E-10 | 175799 | 41.596 |
| 115 | rs11987861 | G | A | 0.009 | -0.048 | 8 | 1.971E-08 | 175799 | 31.287 |
| 116 | rs6471815 | G | A | 0.008 | -0.051 | 8 | 2.711E-11 | 175799 | 44.158 |
| 117 | rs10957321 | A | G | 0.007 | 0.049 | 8 | 3.665E-11 | 175799 | 43.848 |
| 118 | rs11779128 | T | C | 0.008 | -0.043 | 8 | 3.978E-08 | 175799 | 30.470 |
| 119 | rs10103330 | A | T | 0.009 | 0.063 | 8 | 1.450E-11 | 161405 | 45.601 |
| 120 | rs73219806 | A | C | 0.009 | 0.071 | 8 | 2.711E-14 | 175799 | 57.851 |
| 121 | rs4043663 | A | G | 0.009 | -0.058 | 8 | 3.628E-11 | 175799 | 43.688 |
| 122 | rs10086619 | G | A | 0.010 | 0.062 | 8 | 2.972E-10 | 175799 | 39.509 |
| 123 | rs498591 | T | A | 0.011 | 0.059 | 9 | 1.361E-08 | 175799 | 32.106 |
| 124 | rs505061 | A | C | 0.007 | 0.050 | 9 | 1.286E-11 | 175799 | 45.653 |
| 125 | rs10985811 | C | T | 0.009 | 0.053 | 9 | 9.334E-09 | 175799 | 32.841 |
| 126 | rs6479494 | G | A | 0.010 | 0.058 | 9 | 9.568E-09 | 175799 | 32.891 |
| 127 | rs13293831 | T | C | 0.010 | 0.059 | 9 | 8.725E-09 | 175799 | 32.926 |
| 128 | rs2381411 | C | T | 0.008 | 0.043 | 9 | 2.423E-08 | 175799 | 30.901 |
| 129 | rs4339716 | G | T | 0.010 | 0.053 | 9 | 4.033E-08 | 175799 | 29.961 |
| 130 | rs72761691 | C | A | 0.011 | 0.062 | 9 | 2.564E-08 | 175799 | 31.095 |
| 131 | rs61857878 | T | A | 0.010 | -0.061 | 10 | 1.837E-10 | 175799 | 40.514 |
| 132 | rs7902292 | C | T | 0.009 | -0.050 | 10 | 4.432E-08 | 175799 | 30.074 |
| 133 | rs113899647 | T | C | 0.016 | 0.090 | 10 | 3.904E-08 | 175799 | 30.354 |
| 134 | rs7893279 | G | T | 0.012 | -0.100 | 10 | 6.545E-16 | 175799 | 65.441 |
| 135 | rs12571643 | A | G | 0.011 | -0.113 | 10 | 1.246E-23 | 175799 | 100.883 |
| 136 | rs17731 | A | G | 0.008 | 0.059 | 10 | 2.945E-14 | 175799 | 58.515 |
| 137 | rs2279311 | C | T | 0.009 | 0.048 | 10 | 4.115E-08 | 175799 | 29.757 |
| 138 | rs12363019 | A | T | 0.008 | 0.052 | 11 | 5.906E-11 | 175799 | 42.901 |
| 139 | rs708228 | T | C | 0.008 | 0.049 | 11 | 3.992E-09 | 175799 | 34.571 |
| 140 | rs3016382 | A | C | 0.008 | -0.046 | 11 | 1.345E-09 | 175799 | 36.476 |
| 141 | rs10767735 | C | G | 0.008 | -0.050 | 11 | 7.150E-11 | 175799 | 42.840 |
| 142 | rs2902858 | C | T | 0.010 | 0.088 | 11 | 2.690E-18 | 175799 | 76.085 |
| 143 | rs72943392 | C | G | 0.009 | 0.050 | 11 | 4.711E-08 | 175799 | 30.075 |
| 144 | rs77502336 | C | G | 0.008 | 0.055 | 11 | 6.942E-12 | 175799 | 46.587 |
| 145 | rs11219774 | C | A | 0.010 | -0.069 | 11 | 7.788E-12 | 175799 | 46.803 |
| 146 | rs61902811 | A | G | 0.009 | -0.067 | 11 | 5.332E-15 | 175799 | 61.604 |
| 147 | rs11222406 | G | A | 0.007 | -0.041 | 11 | 3.646E-08 | 175799 | 30.251 |
| 148 | rs4936216 | T | C | 0.010 | -0.077 | 11 | 6.281E-14 | 175799 | 56.328 |
| 149 | rs3017989 | C | T | 0.008 | 0.047 | 11 | 1.368E-09 | 175799 | 36.922 |
| 150 | rs302317 | A | G | 0.008 | -0.052 | 12 | 7.869E-11 | 175799 | 41.854 |
| 151 | rs61920311 | C | A | 0.008 | -0.046 | 12 | 8.700E-09 | 175799 | 33.056 |
| 152 | rs578470 | C | T | 0.008 | 0.046 | 12 | 8.418E-09 | 175799 | 33.462 |
| 153 | rs4766428 | T | C | 0.008 | 0.068 | 12 | 8.110E-17 | 175799 | 68.770 |
| 154 | rs61937595 | T | C | 0.016 | -0.120 | 12 | 1.531E-14 | 175799 | 58.974 |
| 155 | rs2686386 | T | C | 0.009 | -0.055 | 12 | 6.279E-09 | 175799 | 33.858 |
| 156 | rs1790135 | T | C | 0.008 | -0.084 | 12 | 6.909E-24 | 175799 | 100.485 |
| 157 | rs4298967 | G | A | 0.009 | -0.083 | 12 | 1.279E-21 | 175799 | 92.029 |
| 158 | rs1526803 | G | C | 0.008 | -0.047 | 12 | 1.661E-08 | 175799 | 32.160 |
| 159 | rs61924144 | C | A | 0.013 | 0.079 | 12 | 4.147E-09 | 175799 | 34.406 |
| 160 | rs6538539 | T | G | 0.007 | -0.045 | 12 | 9.131E-10 | 175799 | 37.140 |
| 161 | rs1426371 | A | G | 0.009 | 0.052 | 12 | 2.674E-09 | 175799 | 35.046 |
| 162 | rs11619756 | A | G | 0.008 | -0.051 | 13 | 2.040E-10 | 175799 | 40.319 |
| 163 | rs9569795 | C | T | 0.010 | 0.064 | 13 | 5.544E-10 | 175799 | 38.762 |
| 164 | rs9597388 | A | G | 0.010 | -0.063 | 13 | 8.978E-11 | 175799 | 41.648 |
| 165 | rs9545047 | C | A | 0.008 | -0.055 | 13 | 4.123E-12 | 175799 | 48.652 |
| 166 | rs61973697 | A | G | 0.010 | 0.055 | 13 | 1.566E-08 | 175799 | 32.033 |
| 167 | rs650520 | T | G | 0.008 | 0.047 | 13 | 5.653E-09 | 161405 | 33.951 |
| 168 | rs12877581 | C | G | 0.009 | 0.052 | 13 | 2.441E-08 | 175799 | 31.340 |
| 169 | rs10148671 | C | T | 0.008 | 0.047 | 14 | 4.139E-09 | 175799 | 34.950 |
| 170 | rs12883788 | T | C | 0.008 | 0.054 | 14 | 1.297E-12 | 175799 | 50.865 |
| 171 | rs1676062 | A | C | 0.008 | -0.045 | 14 | 1.018E-08 | 175799 | 32.836 |
| 172 | rs10873538 | G | T | 0.008 | 0.059 | 14 | 6.142E-14 | 175799 | 56.535 |
| 173 | rs1540840 | C | G | 0.009 | -0.057 | 14 | 9.654E-11 | 175799 | 41.808 |
| 174 | rs2190873 | C | T | 0.007 | -0.063 | 14 | 2.124E-17 | 175799 | 72.249 |
| 175 | rs11632947 | T | C | 0.008 | 0.055 | 15 | 1.639E-13 | 175799 | 54.556 |
| 176 | rs637137 | A | T | 0.008 | -0.065 | 15 | 8.314E-15 | 175799 | 60.433 |
| 177 | rs56282503 | C | T | 0.009 | 0.056 | 15 | 6.628E-10 | 175799 | 38.306 |
| 178 | rs2929278 | T | C | 0.009 | -0.057 | 15 | 6.006E-11 | 175799 | 42.775 |
| 179 | rs11854073 | A | G | 0.008 | -0.048 | 15 | 3.659E-09 | 175799 | 35.116 |
| 180 | rs10906984 | C | A | 0.008 | -0.049 | 15 | 8.035E-11 | 175799 | 41.746 |
| 181 | rs176648 | G | T | 0.008 | -0.046 | 15 | 1.385E-09 | 175799 | 36.948 |
| 182 | rs4702 | A | G | 0.008 | -0.079 | 15 | 1.757E-23 | 175799 | 99.990 |
| 183 | rs9925915 | C | G | 0.008 | -0.057 | 16 | 2.508E-14 | 175799 | 57.768 |
| 184 | rs11862968 | G | C | 0.009 | 0.055 | 16 | 4.715E-09 | 175799 | 34.473 |
| 185 | rs12925872 | T | C | 0.009 | 0.067 | 16 | 1.869E-13 | 175799 | 54.703 |
| 186 | rs11076631 | G | A | 0.008 | -0.051 | 16 | 1.329E-09 | 175799 | 37.024 |
| 187 | rs8048039 | T | A | 0.008 | -0.051 | 16 | 1.979E-10 | 175799 | 39.950 |
| 188 | rs9302397 | A | G | 0.009 | 0.049 | 16 | 1.489E-08 | 175799 | 32.201 |
| 189 | rs11647188 | G | A | 0.008 | -0.044 | 16 | 1.077E-08 | 175799 | 32.949 |
| 190 | rs12950148 | G | A | 0.010 | 0.056 | 17 | 1.918E-08 | 175799 | 31.478 |
| 191 | rs959071 | T | C | 0.011 | -0.075 | 17 | 4.048E-12 | 175799 | 47.974 |
| 192 | rs4793888 | A | G | 0.009 | 0.057 | 17 | 1.124E-09 | 175799 | 37.283 |
| 193 | rs4293 | A | G | 0.008 | 0.043 | 17 | 2.210E-08 | 175799 | 31.719 |
| 194 | rs11263770 | A | G | 0.008 | -0.043 | 17 | 1.743E-08 | 175799 | 31.418 |
| 195 | rs2696466 | G | A | 0.008 | -0.052 | 17 | 1.014E-10 | 175799 | 41.687 |
| 196 | rs12943566 | G | A | 0.008 | 0.049 | 17 | 7.851E-10 | 175799 | 37.997 |
| 197 | rs75329315 | G | T | 0.013 | -0.080 | 17 | 9.376E-10 | 175799 | 37.584 |
| 198 | rs17512480 | A | T | 0.024 | 0.175 | 18 | 5.604E-13 | 165729 | 52.056 |
| 199 | rs7238071 | G | A | 0.008 | 0.059 | 18 | 2.478E-13 | 175799 | 53.241 |
| 200 | rs35360904 | G | T | 0.008 | -0.056 | 18 | 5.404E-14 | 175799 | 56.151 |
| 201 | rs11083369 | T | G | 0.008 | 0.061 | 18 | 3.054E-13 | 175799 | 53.663 |
| 202 | rs715170 | T | C | 0.009 | -0.061 | 18 | 5.112E-12 | 175799 | 48.052 |
| 203 | rs72980087 | A | G | 0.008 | 0.061 | 18 | 1.920E-15 | 175799 | 62.766 |
| 204 | rs72986630 | T | C | 0.017 | 0.108 | 19 | 6.313E-10 | 175799 | 38.381 |
| 205 | rs322124 | G | C | 0.009 | 0.052 | 19 | 4.534E-09 | 175799 | 34.274 |
| 206 | rs8101499 | G | A | 0.008 | -0.059 | 19 | 1.773E-14 | 175799 | 59.115 |
| 207 | rs3810450 | C | T | 0.016 | -0.089 | 19 | 1.374E-08 | 175799 | 32.280 |
| 208 | rs7251 | G | C | 0.008 | -0.058 | 19 | 3.793E-13 | 175799 | 52.931 |
| 209 | rs758749 | T | C | 0.011 | 0.062 | 19 | 1.485E-08 | 175799 | 32.047 |
| 210 | rs2387414 | C | G | 0.008 | 0.049 | 19 | 1.396E-09 | 175799 | 36.440 |
| 211 | rs1006945 | T | G | 0.007 | 0.060 | 20 | 3.300E-16 | 175799 | 65.962 |
| 212 | rs11696755 | C | T | 0.010 | 0.063 | 20 | 2.610E-10 | 175799 | 39.937 |
| 213 | rs8134737 | T | A | 0.008 | 0.047 | 21 | 9.483E-10 | 175799 | 37.764 |
| 214 | rs229362 | A | G | 0.010 | -0.053 | 21 | 2.324E-08 | 175799 | 31.248 |
| 215 | rs1058167 | G | A | 0.008 | 0.054 | 22 | 4.887E-11 | 175799 | 42.963 |
| 216 | rs732381 | T | A | 0.009 | 0.066 | 22 | 1.171E-13 | 175799 | 54.657 |
| 217 | rs6010045 | C | T | 0.008 | 0.046 | 22 | 1.603E-08 | 175799 | 31.976 |

Supplementary Table 10 IVs of sleep apnea.

|  | SNP | effect_allele | other_allele | se | beta | chr | pval | F |
| --- | --- | --- | --- | --- | --- | --- | --- | --- |
| 1 | rs10928560 | T | C | 0.016 | -0.088 | 2 | 2.802E-08 | 30.880 |
| 2 | rs4837016 | A | G | 0.013 | -0.071 | 9 | 1.527E-08 | 31.900 |
| 3 | rs10507084 | T | C | 0.016 | 0.109 | 12 | 2.797E-11 | 44.308 |
| 4 | rs9937053 | A | G | 0.013 | 0.102 | 16 | 4.319E-16 | 66.586 |
| 5 | rs142006783 | C | T | 0.033 | 0.178 | 16 | 4.813E-08 | 29.731 |


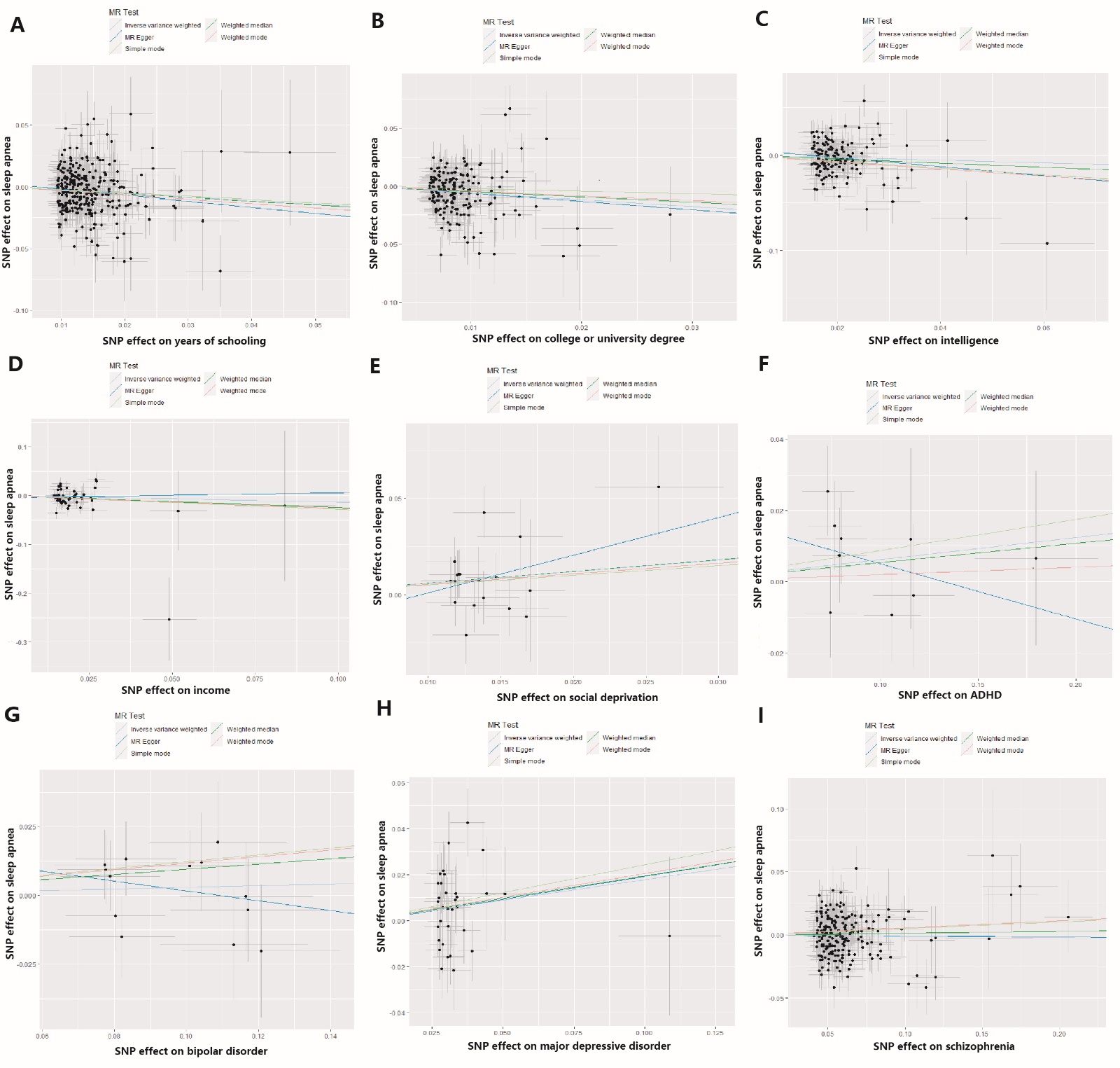


Supplementary Figure 1. The scatter plots for causal effect from mental health and socioeconomic status to sleep apnea. A. Years of schooling. B. College or university degree. C. Intelligence. D. Income. E. Social deprivation. F. Attention-deficit hyperactivity disorder (ADHD). G. Bipolar disorder. H. Major depressive disorder. I. Schizophrenia.


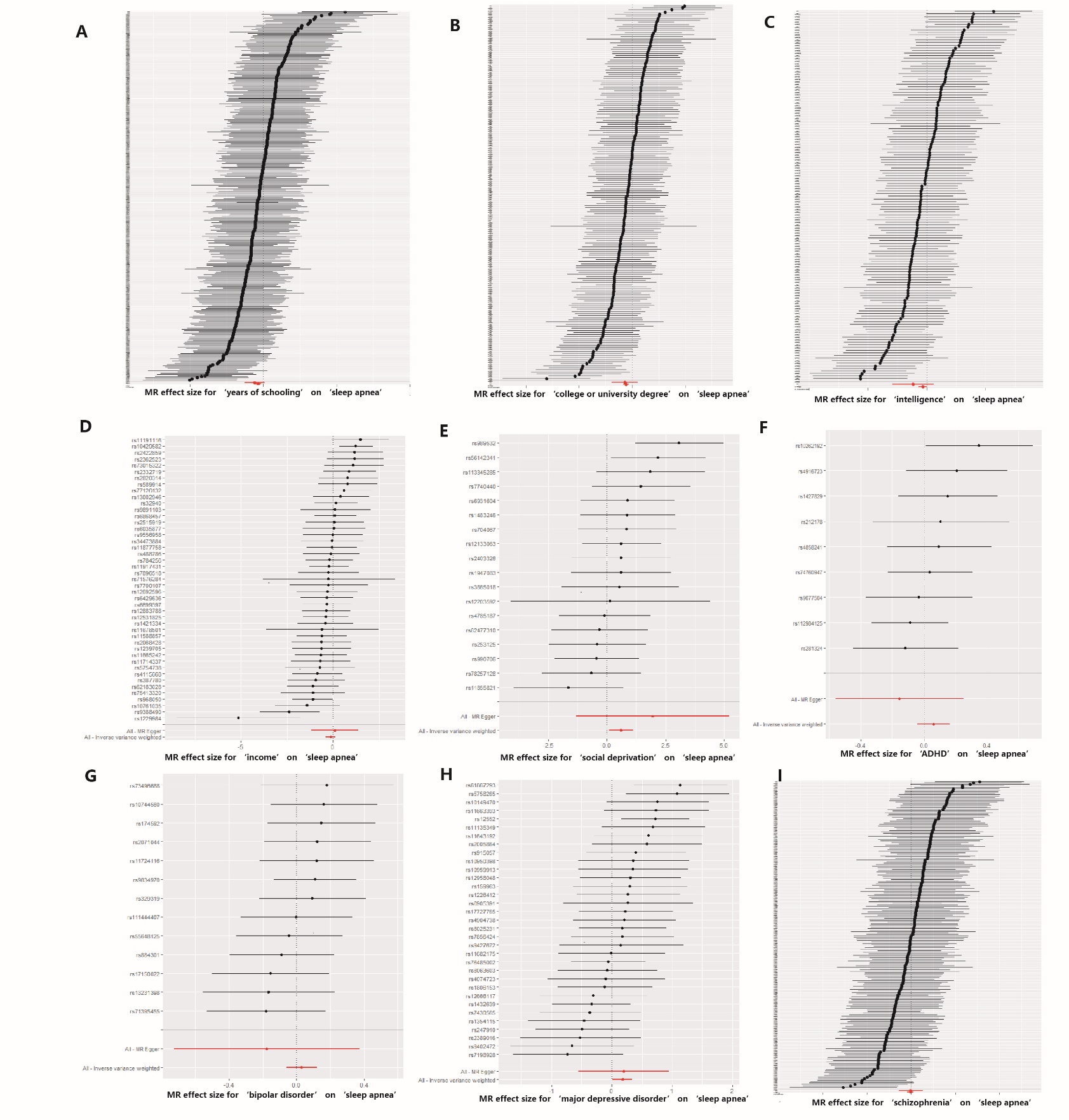


Supplementary Figure 2. The forrest plots for causal effect from mental health and socioeconomic status to sleep apnea. A. Years of schooling. B. College or university degree. C. Intelligence. D. Income. E. Social deprivation. F. Attention-deficit hyperactivity disorder (ADHD). G. Bipolar disorder. H. Major depressive disorder. I. Schizophrenia.


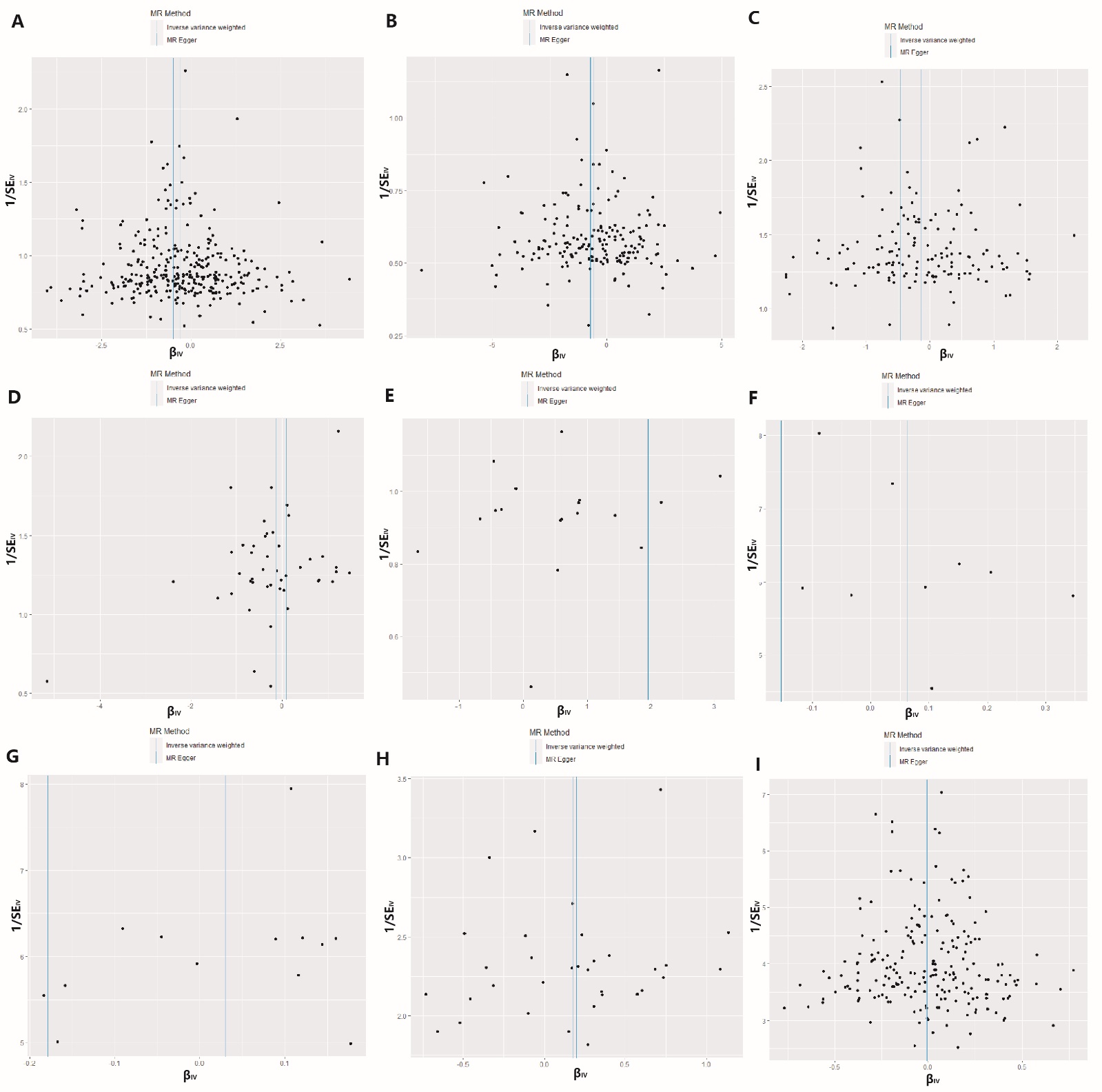


Supplementary Figure 3. The funnel plots for causal effect from mental health and socioeconomic status to sleep apnea. A. Years of schooling. B. College or university degree. C. Intelligence. D. Income. E. Social deprivation. F. Attention-deficit hyperactivity disorder (ADHD). G. Bipolar disorder. H. Major depressive disorder. I. Schizophrenia.
